# Photoexcitation Processes in Oligomethine Cyanine Dyes for Dye-Sensitized Solar Cells—Synthesis and Computational Study

**DOI:** 10.3390/nano10040662

**Published:** 2020-04-02

**Authors:** Corneliu I. Oprea, Petre Panait, Zahraa M. Essam, Reda M. Abd El-Aal, Mihai A. Gîrțu

**Affiliations:** 1 Department of Physics and Electronics, Ovidius University of Constanța, 900527 Constanța, Romania; cornel.oprea@univ-ovidius.ro; 2 Doctoral School, Faculty of Physics, University of Bucharest, 077125 Bucharest, Romania; p_panait@yahoo.com; 3 Department of Chemistry, Suez University, 43511 Suez, Egypt; zahraa.essam@suezuniv.edu.eg

**Keywords:** oligomethine cyanine-based dyes, density functional theory, optical spectra, titanium dioxide cluster, dye-sensitized solar cells

## Abstract

We report density functional theory (DFT) calculations of three newly synthesized oligomethine cyanine-based dyes as potential TiO_2_-sensitizers in dye-sensitized solar cells. The three dyes have π-symmetry and the same acceptor side, terminating in the carboxylic anchor, but they differ through the π-bridge and the donor groups. We perform DFT and time-dependent DFT studies and present the electronic structure and optical properties of the dyes alone as well as adsorbed to the TiO_2_ nanocluster, to provide some predictions on the photovoltaic performance of the system. We analyze theoretically the factors that can influence the short circuit current and the open circuit voltage of the dye-sensitized solar cells. We examine the matching of the absorption spectra of the dye and dye-nanocluster system with the solar irradiation spectrum. We display the energy level diagrams and discuss the alignment between the excited state of the dyes and the conduction band edge of the oxide as well as between the redox level of the electrolyte and the ground state of the dyes. We determine the electron density of the key molecular orbitals and analyze comparatively the electron transfer from the dye to the semiconducting substrate. To put our findings in the right perspective we compare the results of our calculations with those obtained for a coumarin-based dye used in fabricating and testing actual devices, for which experimental data regarding the photovoltaic performance are available.

## 1. Introduction

Among renewable energy sources, photovoltaics have demonstrated the most rapid cost reduction over the past decade [1,2], decreasing by a factor of more than 15, led by technology improvements, particularly in silicon wafer-based cells, which account for 95% of the market in 2018 [3]. Under these circumstances, dye-sensitized solar cells (DSSC) [4], which offer several advantages, such as low fabrication costs, transparency and flexibility complexes [5,6], may represent an attractive choice only in niche applications, for example in affordable low power generation in urban areas, particularly in power generating windows [7,8,9].

The photovoltaic conversion efficiency of the solar cells depends strongly on the dye and electrolyte used [10], reaching 11% for DSSCs with iodide/triiodide electrolyte and Ru(II)-polypyridyl complexes [11,12] and, more recently, 11.5% with porphyrin dyes [13]. By replacing the iodine electrolyte with cobalt-based complexes, the efficiency was increased to 12% [14], or even higher [15,16,17,18,19], with the use of organic dyes.

The criteria that should be fulfilled by a dye to be an efficient solar cell sensitizer include [10,20]: adsorption at the interface with TiO_2_, matching of the absorption with the solar irradiation spectrum, energy level alignment between the excited and ground states of the dye and the conduction band and the redox level of the electrolyte, respectively, etc. These criteria need to be optimized for performance enhancement. The desirable processes in DSSCs are [21,22,23] light harvesting, charge injection from the dye into the oxide, charge diffusion through the semiconducting nanocrystals and dye regeneration by charge transfer from the electrolyte, whereas detrimental processes include electron back transfer to the dye, charge interception by the electrolyte and loss of photoelectrons through luminescence.

The metal free organic sensitizers that allowed the fabrication of devices with high photovoltaic conversion efficiencies have in common the π-symmetry of the molecule, consisting in essence of three parts: an electron donating group, a π-bridge and a charge acceptor (D−π−A) [10,24,25,26]. The structure of the three building blocks of the dyes has a direct influence on the photovoltaic performance of the devices, affecting the light harvesting properties, the energy level alignment as well as the push→pull effects and the charge transfer [10,20,22]. Consequently, small changes in the structure of these building blocks, such as the extension of the length of the conjugated backbone, the substitution of various atoms and groups in any of the three parts, the inclusion of various peripheral groups and so on, permit the fine tuning of the optical properties of the dye [18,26,27]. Moreover, it was shown that a rigid geometric structure of the dye is crucial to decrease losses due to vibrational relaxation [19].

In the search for organic dyes to be used as sensitizers in DSSCs, several oligomethine cyanine dyes (OMCD) have been reported [28,29,30,31,32,33]. Common to all reported OMCD dyes was the position of the carboxylic acid anchor group at both ends of the heterocyclic moieties, resulting in symmetric structures. Such systems have the drawback of not being optimized for electron transfer, as they do not have an asymmetric D−π−A structure that favors push→pull effects. The three oligomethine cyanine dyes that we report here are asymmetrical, with only one carboxylic acid anchor group. The dyes have π-symmetry and the same acceptor side, terminating in triphenyl phosphonine at the other end, the differences arising from the various donor groups.

The goal of the present study is twofold: to determine whether the three dyes synthesized are good candidates for photovoltaic applications and to analyze the effect of the different substitutions on the electronic and optical properties of these sensitizers. We use molecular modelling techniques based on density functional theory (DFT) [34,35,36] and time-dependent DFT [37] calculations to perform structure property correlations and verify whether the selected dyes fulfil the criteria mentioned above. Such DFT calculations of dye-TiO2 nanocluster systems have been useful in understanding the DSSC experimental data available for ruthenium-based [38,39], metal-free [40] or natural dyes [41]. Additionally, to place our findings in the proper context we compare the results of our calculations with those obtained for a coumarin-based dye [42], for which experimental data are available [43,44]. Finally, we also comment on the superiority of the more holistic approach, dealing with the dye and the substrate together.

## 2. Materials and Methods

### 2.1. Materials—Synthesis of Oligomethine Cyanine Dyes

**Scheme 1:**
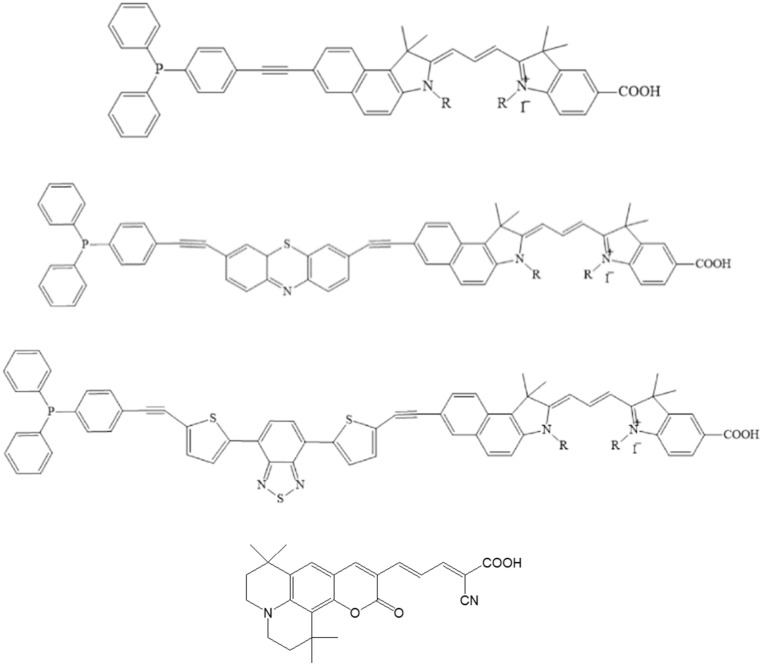
Chemical structures of OMCD1, OMCD2, OMCD3, and NKX-2311, from top down, R = CH_3_, respectively.

The TiO_2_ sensitizers must have an anchoring group for binding to the oxide surface with a significant electronic coupling (orbital overlap). Here, as in most cases [10], the anchor is the –COOH group. For all three dyes, the carboxylic group is in conjugation with a heterocyclic moiety (indolinium), serving the role of an electron acceptor. Common to all dyes is also the polymethine bridge, which is rigidified by benzo[e]indolinium groups. All dyes terminate in triphenyl phosphonine via ethynyl bonds, but the electron donor part is different due to the additional phenothiazine or the benzothiadiazine in between two thiophene groups (see Scheme 1). In making these choices, the expectation was that the absorption spectra of the dyes would be red-shifted by lengthening the conjugated molecule and that different donor groups can shift the ground and excited states fine-tuning the absorption and the energy level alignment. In order to improve solubility in organic solvents and to hinder dye aggregation during immobilization on the semiconductor, the heterocycles were substituted with alkyl groups.

The three oligomethine cyanine-based dyes studied here are displayed in Scheme 1. Additionally a coumarin-based dye was also studied, as it allows for illustrative comparison due to the existing experimental data [43,44]:

(i) 5-carboxy-2-(7-(4-diphenylphospho)phenyl)ethynyl-1,1,3-trimethyl-1H-benzo[e]indol-2(3H)-yliedene)prop-1-ene-1-yl)-5-carboxy-1,3,3,-trimethyl-3H-indolium iodide (**OMCD1**),
(ii) 7-(4-diphenylphosho)phenyl)ethynyl-3-yne-(7(1,1,3-trimethyl-1H-benzo[e]indol-2(3H)-yliedene)prop-1-ene-1-yl)-5-carboxy-1,3,3,-trimethyl-3H-indol-1-ium iodide-phenothiazine 11 (**OMCD2**),
(iii) 4,7-bis(5--(4-diphenylphospho)phenyl)ethynyl-5-yne-thiophene-2,2--diyl)(1,1,3-trimethyl-1H-benzo[e]indol-2(3H)-yliedene)prop-1-ene-1-yl)-5-carboxy-1,3,3,-trimethyl-3H-indol-1-ium iodide-4,7-dithienyl-benzo[c][2,1,3]thiadiazol (**OMCD3**),
(iv) 2-cyano-5-(1,1,6,6-tetramethyl-10-oxo-2,3,5,6-tetrahydro-1H,4H,10H-11-oxa-3a-aza-benzo[de]anthracen-9-yl)-penta-2,4-dienoic acid (**NKX-2311**).

**Scheme 2:**
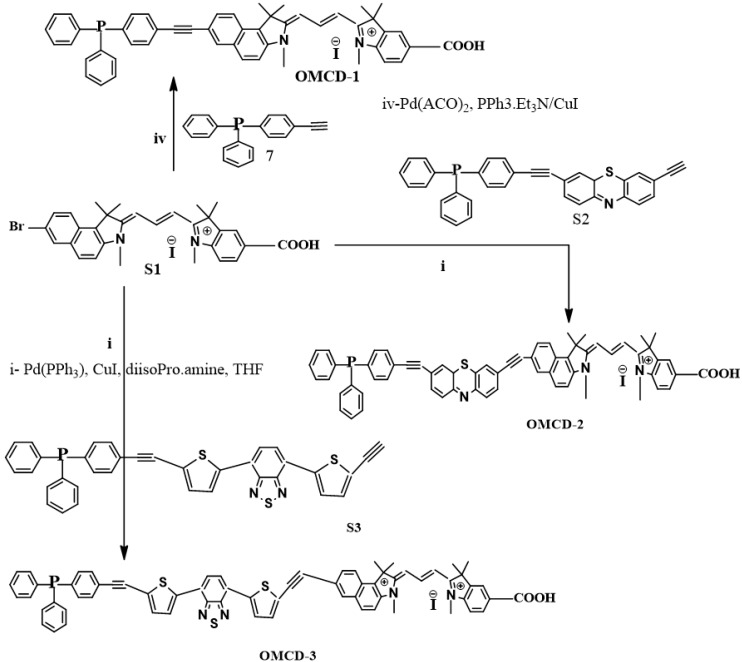
Synthesis of OMCD1, OMCD2, and OMCD3.

The details of the synthesis of the three dyes are given in the Supplementary Materials. Here we only give a general description, as illustrated in Scheme 2. The key starting material for the synthesis of all three dyes is 6-bromo-2-diethylidene 1,3,3-trimethyl-3H-benzo[e]indolium-2yl 1,3,3-trimethyl indolinium-2yl-5-carboxy-iodide, **S1** (see Scheme 2). The reaction of equimolar ratio of **S1** with (4-diphenylphospho)phenyl)ethynyl 7 in the presence of catalyst, Pd(OAc)2/PPh3,CuI/Et3N [45], afforded the OMCD 1 dye. Reaction of equimolar amount of **S1** and 7-(4-diphenylphosho)phenyl)ethynyl-3-yne-phenothiazine, **S2**, according to the literature [46] lead to OMCD 2. Synthesis of OMCD 3 was accomplished using Suzuki and Stille coupling routes [47,48], reacting equimolar ratios of **S1** and 4,7-bis(5--ethynyl)(4-diphenylphospho)phenyl)ethynyl-thiophene-2,2--diyl)-benzo[c] [1,2,5] thiadiazol, **S3**, with stepwise dehydrohalogenation.

Solvents and other chemicals were purchased from commercial sources (Sigma-Aldrich, Taufkirchen, Germany), and used without purification. Unless otherwise stated, all reactions were carried out under inert gas (Argon) in anhydrous solvent. Sodium acetate was dried under high vacuum at 120 °C for 3 h. ^1^H NMR spectra were recorded at 298 K on a 5 mm broadband inverse probe using a Brüker Avance 400 NMR spectrometer (Billerica, MA, USA). Chemical shifts (δ), in ppm, were calibrated to residual solvent peaks (CDCl_3_). In the Supplementary Materials, coupling constants *J* are reported in Hz and for ^1^H NMR data coupling patterns are described as s = singlet, d = doublet, t = triplet, q = quartet, m = multiplet, and br = broad. Mass spectroscopy data were recorded on a Bruker maXis-ESI-Q-TOF mass spectrometer (ESI-QTOF, Billerica, MA, USA) or on a HiRes-ESI IonSpec Varian Ultima-ESI FT-ICR-MS spectrometer (Palo Alto, CA, USA). Elemental analyses for C, H, N, were measured by an apparatus from LECO (Plzeň, Czech Republic). IR spectra were recorded on a Bruker Tensor 27 (Billerica, MA, USA) with ATR configuration. UV-Vis spectra of the dyes in solutions were recorded on a Varian Cary 50 Scan spectrometer (Palo Alto, CA, USA) in a 1.0 cm square cuvette. Characterization data for all three dyes and the intermediate compounds as well as vibrational and nuclear magnetic resonance spectra are reported in the Supplementary Materials.

### 2.2. Computational Details

The structures of the isolated dyes were optimized in neutral forms by density functional theory (DFT) [34,35,36] using the hybrid exchange-correlation functional B3LYP [49,50] and all electron basis sets for all atoms via DZVP [51]. For the electronic structure, single-point calculations were performed using the hybrid B3LYP functional with the same basis set. In the case of isolated dye molecules, extra polarization functions required for more accurate electronic densities were included via the DZVP basis sets. Singlet-to-singlet electronic transitions were calculated by time-dependent-DFT (TD-DFT) [37], their number varying from 10 to 200 depending on the size of the system.

In a previous study, following the work of Persson et al. [52] and De Angelis et al. [53] we showed that (TiO_2_)*_n_* clusters with *n* less than 24 cannot properly describe the complex dye-oxide systems [54]. It was concluded that the *n* = 24 cluster was the smallest one that can be used well for qualitative calculations, particularly when the adsorbed molecule is rigid, as it is the case here [54,55].

The model clusters used has a slight deviation from the TiO_2_ stoichiometry which circumvents the problem of the surface states in the gap. We introduced H atoms or –OH groups to terminate the peripheral dangling bonds, resulting in the Ti_24_O_50_H_4_, and we showed that the presence of the four H atoms does not affect the nature of the electronic spectrum [54,55].

The structures of the dyes adsorbed onto the Ti_24_O_50_H_4_ cluster were optimized by DFT using the B3LYP functional and the effective core potentials LANL2DZ basis sets [56]. For the electronic structure of the adsorbed dyes, single-point calculations were performed using the DZVP basis sets [51].

To ensure the π conjugation over the nitrogen atoms in the cyanine constituents the oligomethine cyanine-based dyes have to be cationic species. To ensure the overall neutrality during the calculation, we placed a negative iodine ion in the vicinity of the cyanine groups. The presence of iodine in the DSSCs justifies this modelling and computational artifice.

The solvent effect was accounted for by employing the polarizable continuum model (PCM) [57,58], which treats the solvent as a homogeneous dielectric medium. The cavity used in the PCM calculation was built from spheres cantered on heavy nuclei, based on the United Atom for Hartree–Fock procedure described in [57]. All calculations were performed with the GAUSSIAN09 quantum chemistry package [59].

### 2.3. Quantities Relevant to Device Performance

The key indicator of the device performance is the photovoltaic conversion efficiency, *η*, defined as the ratio between the maximum electric power delivered to the external load, *P_e,max_*, and the incident optical power, *P_o,inc_* [10]. The efficiency can be expressed in terms of other important parameters:

(1) $$\eta =\frac{P_{e,max}}{P_{o,inc}}=FF\frac{J_{sc}V_{oc}}{P_{o,inc}}$$

the fill factor (*FF*, which is a good indicator of the quality of the device, affected by series and shunt losses), the short-circuit current density, *J_sc_*, and the open-circuit voltage, *V_oc_*.

The voltage generated under illumination corresponds to the difference between the electrochemical potential of the electron at the two contacts, which for DSSCs is the difference between the quasi-Fermi level of the electrons at the TiO_2_/transparent conducting oxide, $E_{F}^{n}$, and the redox level of the electrolyte/platinized TCO interface, *E_redox_* [4,10,20]:

(2) $$eV_{oc}=E_{F}^{n}-E_{redox}=E_{CB}+\beta k_{B}Tln\left(\frac{n_{c}}{N_{CB}}\right)-E_{redox}$$

The Fermi level at the photoelectrode is approximated by the energy of the conduction band edge, *E*_CB_, from which it can depart, depending on the ratio between the density of electrons, *n_c_*, and the density of states, *N*_CB_, in the conduction band of the TiO_2_ semiconductor. In Equation (2) *e* is the elementary charge and *k*_B_*T* the thermal energy at room temperature [10,60,61]. The *β* parameter is unitary in the ideal case of a defect-free semiconductor but less than 1 in the case of mesoporous TiO_2_, with trap electronic states [62]. Furthermore, some authors make an adjustment for the shift in the conduction band when the dye is adsorbed on the surface [63,64].

To avoid that a mistake is perpetuated, it should be noted that in the literature there are several reports using a wrong relation to determine *V*_oc_ (see, for instance, References. [65,66,67,68,69,70,71,72,73,74]). In those cases, a confusion is made with the driving force for charge injection from the dye into the semiconductor, $\Delta G_{inj}$, which is calculated as the difference between the energy of the excited state of the dye (lowest unoccupied molecular orbital in DFT calculations), E_dye*_, and the TiO_2_ conduction band edge, *E_CB_* [10,63,75]:

(3) $$\Delta G_{inj}=E_{dye*}-E_{CB}$$

The energy of the excited state of the dye can be determined directly from single point DFT calculations and from TD-DFT calculations, which provide the energy of the singlet-singlet vertical transition (considering the lowest vibrational levels of both states), Δ*E*_0-0_, from the ground state, *E_dye_* [10,62].

(4) $$E_{dye*}=E_{dye}+\Delta E_{0-0}$$

The short-circuit current density can be determined by integrating the incident photon to current conversion efficiency (IPCE), which can be experimentally measured as the ratio between the photocurrent density produced in the external circuit, under monochromatic illumination of the cell, and the photon flux, $\Phi_{ph}$, that strikes the cell [10,76]:

(5) $$J_{sc}=\int ICPE\left(\lambda \right)e\Phi_{ph}\left(\lambda \right)d\lambda$$

or

(6) $$J_{sc}=e\int LHE\left(\lambda \right)\eta_{inj}\left(\lambda \right)\eta_{reg}\eta_{cc}\left(\lambda \right)\Phi_{ph}\left(\lambda \right)d\lambda$$

where *η*_inj_ and *η*_reg_ are the quantum yields for electron injection and dye regeneration, respectively, and *η*_cc_ is the charge collection efficiency, whereas LHE is the light harvesting efficiency

(7) $$LHE\left(\lambda \right)=1-10^{-\sigma \left(\lambda \right)\Gamma }$$

*σ*(*λ*) being the absorption cross section (in units of cm^2^/mol), obtained from the decadic extinction coefficient, *ε*(*λ*) (units of mol^−1^·cm^−1^) by multiplication with 1000 cm^3^, and *Γ* is the number of moles of sensitizer per square centimetre of projected surface area of the film [10,77,78].

We note that the calculation of the short-circuit current density is not trivial, as the functions under the integral in Equation (6) are not easily available. The solar photon flux is accessible [79] and has recently been used [78], although in other reports, the solar photon flux was approximated based on Planck’s blackbody radiation approximation [80,81]. One challenge is to determine theoretically the electron injection, dye regeneration and charge collection efficiencies. The charge collection efficiency, *η*_cc_, can be considered the same for different dyes [82], as it reflects the electron diffusion through TiO_2_ and the charge transfer at the interface with the conducting oxide. The injection efficiency depends on the driving force, *ΔG_inj_*, with claims that for large values it tends to 1 [82]. In many cases, simplifying assumptions are made, for instance, claiming that the maximum photocurrent can be obtained if the efficiencies in Equation (6) were equal to 1 [78]. A more rigorous approach should take into account the various relaxation times of the processes involved [83,84]. In any case, these interface electronic processes have, at best, a weak dependence on the wavelength of the photon and can be left out of the integral. In contrast, the LHE depends on the wavelength of the incident solar light. However, despite this strong variation, in many cases it is approximated with its value at the maximum absorption peak [80], which leads to significant overestimations. A more careful analysis of the LHE ought to take into account both the spectral dependence, through the extinction coefficient, and the surface concentration of the dye. Further approximations are needed, as the extinction coefficient of the dye adsorbed onto the oxide is not identical with that determined in solution, whereas the surface concentration requires knowledge of the porosity of the film of nanocrystalline TiO_2_.

A simpler and more intuitive measure of the light harvesting properties of a dye is offered by the matching between its absorption spectrum and the solar irradiance spectrum. Calculating the area under the curve obtained by multiplying the calculated absorption spectrum of the dye and the standard global total spectral irradiance:

(8) $$SMC=\int \varepsilon \left(\lambda \right)\Phi_{ph}\left(\lambda \right)d\lambda$$

we find the overlap integral representing the spectral matching coefficient.

## 3. Results

This section is divided in two parts, referring first to the isolated dyes and the second to the dyes bound to the TiO_2_ nanocluster.

### 3.1. Isolated Dye—Structural, Optical and Electronic Properties

**Figure 1:**
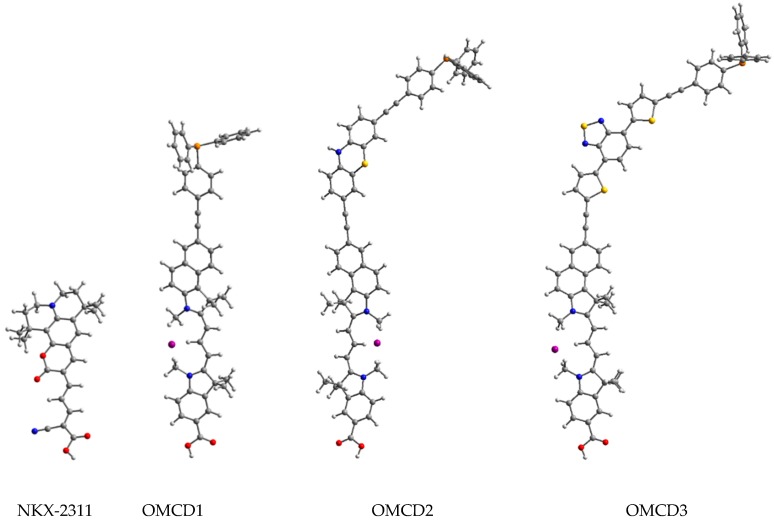
Optimized structure of the dyes in their neutral form, calculated by DFT at B3LYP/DZVP level in ethanol. Color coding: C—grey, O—red, N—blue, S—yellow, P—orange, I—purple, H—light grey, small sphere.

The structures, optimized by DFT calculations, of the three oligomethine cyanine-based dyes and the reference coumarin-based dye are displayed in Figure 1. Some of the key structural parameters are summarized in Table 1. The first two parameters reported in Table 1 are the distance between the C atom of the –COOH anchor and the adjacent C atom on the indolinium and the <(O-C-O) angle of the anchor. It can be seen that the values are the same for the three compounds. The twist angle <(COOH-ind) between the plane of the anchor and the plane of indolinium is very small for all three compounds; the very good alignment facilitates the delocalization of the π electrons.

All three systems have a backbone of three C atoms connecting the indolinium and benzoindolinium groups. The four successive C-C distances, *r1*(C*_i_*-C*_bi_*), and the total distance, *r*(C*_i_*-C*_bi_*), between the two groups show very similar values, which alternate in the range 1.394 to 1.407 Å. The twist angle <(ind-benzoind), between the planes of the two groups, also shows similar values, varying between 12.2° and 15.5°. The departure from co-planarity is not high but can hinder electron delocalization and the intramolecular charge transfer along the dyes.

The opposite end of the dyes with respect to the –COOH anchor is tied to the benzoindolinium moiety through a –C≡C– group. The distances between adjacent C atoms, *r1*(C*_bi_*-C*_r_*), and the distance between the end-connecting C atoms, *r*(C*_bi_*-C*_r_*), show some slight differences among dyes, the shortest inter group distance being recorded for OMCD3. The twist angle <(benzoind-ring), between the planes of the two groups, display significant differences, with values of 2.5°, 21.1°, and 13.6° for the tree dyes respectively. Finally, the distances between the I atom and the N atom on the indolinium group, *r*(I-N*_i_*), vary about 5 Å by less than 4%.

**Figure 2:**
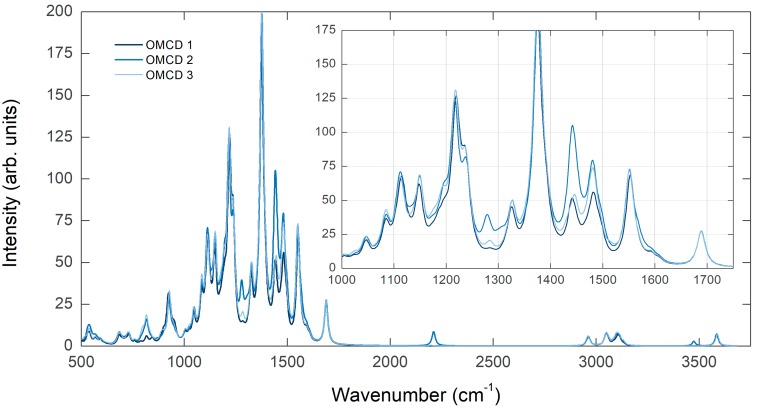
Vibration spectra of the three dyes, OMCD1, OMCD2, OMCD3 calculated by DFT at B3LYP/DZVP level. The spectral lines were convoluted with Lorentz distributions of 20 cm^−1^ linewidth at half maximum. The inset shows a detail of the fingerprint region.

Vibration spectra calculations performed on the optimized geometries provided only real frequencies, indicating that the computed structures are stable, obtained at a true energy minimum. The calculated vibration spectra of the three dyes are shown in Figure 2. The value of the scaling factor used for the vibration frequencies was 0.969 [85]. This value was chosen to adjust the wavenumber of the calculated –CH_3_ symmetric stretch at 1370 cm^−1^, which is the most intense band in the experimental data (reported in the Supplementary Materials).

The three spectra display some similarities, with high wavenumber O–H modes of carboxyl groups, just above 3585 cm^−1^, close to the broad experimental bands observed at about 3380 cm^−1^, and of C≡C triple bonds stretches at 2210 cm^−1^, near those measured, at 2150 cm^−1^. The vibration modes found at 1690 cm^−1^ correspond to the observed ones at 1660 cm^−1^ and 1670 cm^−1^ for C–C bond in the anchor. The second highest calculated peak is at 1215 cm^−1^, is a rocking of the C–H bonds along the backbone, measured at about 1225 cm^−1^. Other bands in the fingerprint region are at about 1550 cm^−1^ corresponding to the C=C stretches along the backbone observed at about 1585 cm^−1^.

**Figure 3:**
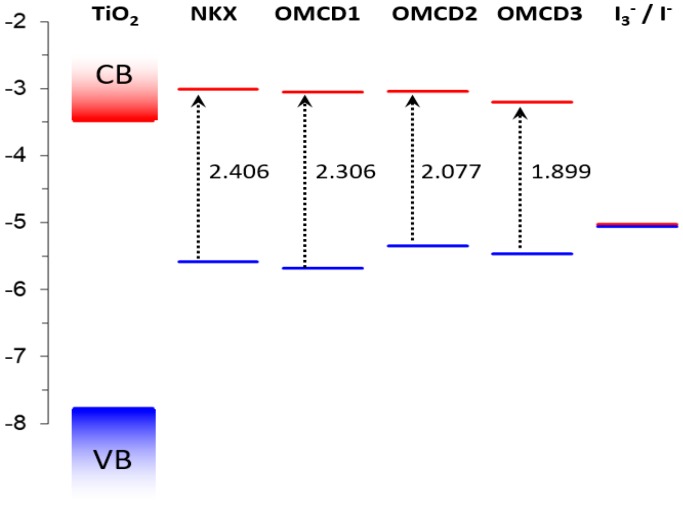
Energy diagram showing the ground state (blue) and the excited state (red) energies calculated in solution by DFT and time-dependent (TD)-DFT (B3LYP/DZVP) methods for the TiO_2_, NKX-2311, OMCD1, OMCD2, OMCD3 molecules and the redox level of the I_3_^−^/I^−^. The values represent the lowest singlet-to-singlet transition, Δ*E*_0-0_, in eV.

The possibility of charge injection from the dye to the semiconducting oxide as well as of the regeneration of the dye by charge transfer from the electrolyte, can be studied by DFT calculations, looking at the energy level alignment between the dye, the substrate and the electrolyte [38,40]. For that purpose, Figure 3 displays the energy of the ground and the excited states of the dyes, together with the energies of the valence (VB) and conduction (CB) band edges of TiO_2_ and the redox level of the I_3_^−^/I^−^ electrolyte. The energy of the excited state can be determined directly from the DFT calculation (which provides the lowest unoccupied molecular orbital, LUMO) and as the sum between the energy of the ground state (highest occupied molecular orbital, HOMO) and the energy of the lowest singlet-to-singlet transition, obtained from TD-DFT calculations (see Equation (4)). The former tends to overestimate the gap, whereas the latter was shown to give a better estimate of the excited state energy [53].

From Figure 3 it can be seen that for all dyes the excited state lies above the conduction band edge, allowing for the transfer of the photoelectron from the dye to the semiconductor. Additionally, as the HOMOs lie below the redox level of the electrolyte, the dye regeneration through electron transfer from the electrolyte is allowed for all dyes.

The driving force for the electron injection from the dye into the semiconductor, ${\Delta G}_{\mathrm{inject}}$, is estimated by the energy difference between the excited state of the dye and the conduction band edge of the oxide, according to Equation (3). In the cases studied here, the driving forces are 0.47 eV for NKX-2311, 0.43 eV for OMCD1, 0.44 eV for OMCD2 and 0.28 eV for OMCD3.

The usual view is that the injection rate increases with the driving force [86,87]. However, other studies claimed that a driving force for electron transfer which is too large may become detrimental, one possible cause being a smaller overlap between the excited state of the dye and the state of corresponding energy on the substrate [42,44]. For the four dyes studied here the driving forces are comparable and, although inclined to state that OMCD1 and OMCD2 are superior dyes, we stress that the driving force is not the only factor influencing the charge injection.

**Figure 4:**
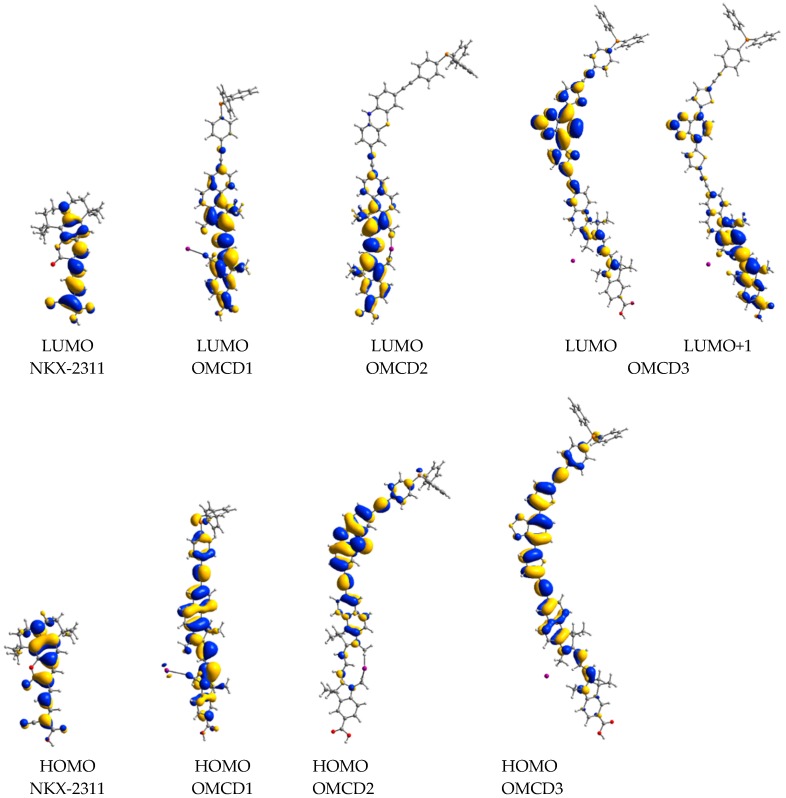
Isodensity surfaces (0.03 e/bohr^3^) of the key molecular orbitals of the isolated dyes, calculated at the DFT/B3LYP/DZVP level in ethanol.

To further explore the likelihood of the charge injection process, it is useful to also look at the push→pull effects, as reflected by the electron densities of the main molecular orbitals (Figure 4) and by the contribution to the electron density of the various building blocks (Table 2). For the oligomethine cyanine-based dyes we took into consideration as acceptor unit the carboxyl anchor and the indolinium group, the π-bridge entails the benzoindolinium group, whereas the donor units differ from one dye to another. For the NKX-2311 dye, we considered that the donor unit consists of the coumarin-quinolizine groups, whereas the acceptor units consist of the cyanoacetic acid moiety, bridged by a π conjugated backbone.

The values reported in Table 2 and the electron densities displayed in Figure 4 show a push→pull effect for OMCD1 and OMCD2, as well as for the reference dye, NKX-2311. For these dyes, in the ground state the electron density is delocalized mostly over the donor unit and the bridge, whereas in the excited state the charge is moved toward the acceptor unit. In contrast, for OMCD3 the charge of the LUMO is mostly localized on the bridge, the push→pull effect being more obvious for the following level, LUMO+1, which lies higher in energy. Under these circumstances, the push→pull effects are significant for OMCD1 and OMCD2, comparable with the reference dye.

**Figure 5:**
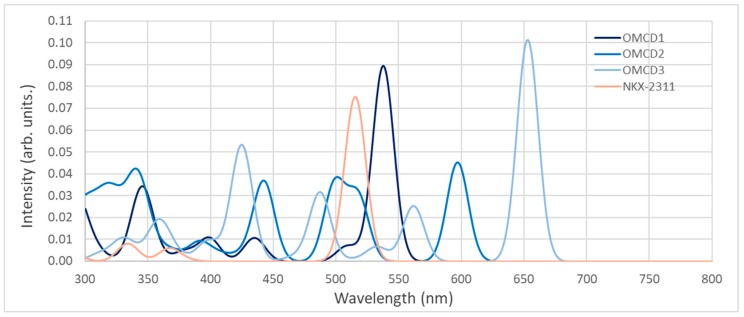
Theoretical UV-Vis spectra by TD-DFT calculation at B3LYP/DZVP level for the dyes in ethanol. The spectral lines were convoluted with Gaussian distributions of 20 nm linewidth at half maximum.

One of the most important requirements for a dye, to be used in DSSCs, is to have an absorption spectrum matching the solar irradiation spectrum. Therefore, we calculated by TD-DFT the UV-Vis-simulated absorption spectra (see Figure 5) in ethanol solvent for all four dyes, using a continuum model (PCM [57,58]).

The calculated visible absorption peaks for OMCD1 are located at 538 nm, 435 nm and 399 nm, the first band being the most intense, corresponding to a transition from the highest occupied to the lowest unoccupied molecular orbitals (HOMO→LUMO). In the UV the transitions, situated at 344, 294 and 279 nm, are more complex connecting states below the HOMO with states above the LUMO. For OMCD2 the peaks are shifted to higher wavelengths: in the visible region at 597 nm, 518 nm, 499 nm and 442 nm, whereas in the UV there are three groups, around 340 nm, 320 nm and 305 nm. The most intense is the first band, again a HOMO→LUMO transition. The most important absorption peaks of OMCD3 are located at even higher wavelengths, at 653 nm, 562 nm, and 425 nm. The highest transitions in the UV are at 398 and 357 nm.

For comparison, the main peak for NKX-2311 is located at 515 nm, the other being in the UV, at 367 nm 334 nm. This is in accord with the experimental value of 504 nm [43]. Details regarding the transitions, particularly the oscillator strength and the major contributions, can be found in the Supplementary Materials. Here, we note that the highest wavelength bands are between the HOMO and LUMO states and all these transitions have a π → π* nature, as it can be seen from Figure 4.

To compare the matching of the UV-Vis absorption bands with the solar spectrum we calculated the area under the curves obtained by multiplying the calculated spectra of the dyes and the standard global total spectral irradiance (in W/sm/nm) on a 37° sun-facing tilted surface, AM1.5 G [79]. The results are displayed in Table 3, with details about the spectra being provided in the Supplementary Materials. It can be seen that the best matching is obtained for OMCD3.

### 3.2. Dye on Substrate—Structural, Optical and Electronic Properties

The anchoring of the dye to the TiO_2_ anatase (101) surface affects the electronic coupling and the charge injection into the semiconducting substrate [1,11,56]. Theoretical calculations and experimental studies have shown that for the dyes bearing a carboxylic acid as the anchoring group, the preferred adsorption mode is bidentate bridging, with one proton transferred to a nearby surface oxygen [88].

**Figure 6:**
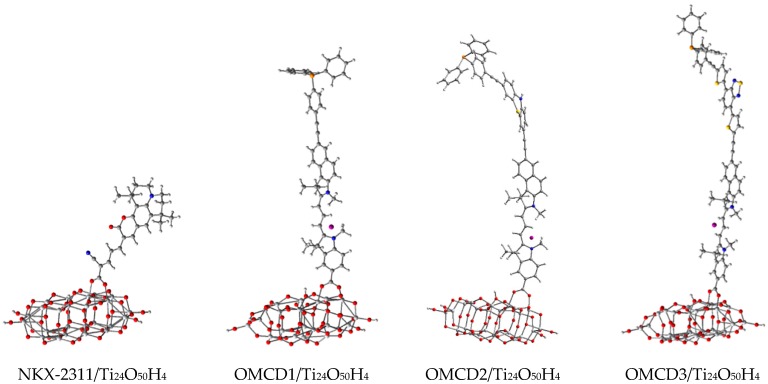
Optimized structure of the dye-oxide systems, calculated by DFT at B3LYP/LANL2DZ level. Colour coding: C—grey, O—red, N—blue, S—yellow, P—orange, I—purple, Ti—light grey, H—light grey small sphere.

As the dyes reported here end with a carboxyl group, a similar behavior is expected. The calculations providing the optimized geometry for the dye adsorbed on the oxide nanocluster confirmed the bidentate bridging type of bonding, with the two oxygen atoms of the carboxyl group binding to adjacent titanium atoms on the surface (Figure 6).

In a previous study [54] we compared geometry parameters for the bare cluster and the cluster with the adsorbed molecule. We noted that for the Ti_24_O_50_H_4_ cluster the average Ti–O distance was 1.876 Å and the standard deviation for that distance 0.100 Å, whereas for the bulk the values are 1.950 Å and 0.022, respectively. The average distances calculated for the oligomethine cyanine dyes and reported in Table 4 vary between 1.876 and 1.899 Å, whereas the standard deviations are in the range 0.104–0.115 Å. These small deviations from the values obtained for the bare clusters indicate that the dyes, despite their size, have only a minor effect on the cluster, the distortion being small, which is typical for rigid adsorbed molecules [54].

Table 4 also presents some key geometry parameters, particularly bond distances at the binding sites and various angles of interest. The Ti–O distances between the oxygen atoms of the anchor and the titanium atoms on the surface are larger than the average distance in the nanocluster as well as in bulk anatase titania. The two bonding Ti–O distances differ systematically, ranging one between 2.067 and 2.085 Å and the other between 1.941 and 1.994 Å. The resulting differences are less than 0.15 Å. These values allow the adsorbed dye to be positioned upright compared to the nanocluster, as it can be seen in Figure 6. Additionally, the values of the dihedral angle between the indolinium plane and the plane of three neighbour Ti atoms on the cluster surface range between 75.6° and 86.4°, favoring a roughly upright position.

The torsion angle of the carboxyl group relative to the indolinium ring plane is relatively small, varying between 2° and 4.8°. The small torsion angle favors the delocalization of the π electron over the acceptor unit of the dye facilitating the charge injection into the substrate.

Other structural data are reported in Table 1, for comparison with the isolated dyes. The distance between the C atom of the carboxyl anchor and the adjacent C atom on the indolinium is slightly but consistently shortened for the dyes bound to the nanocluster, by about 0.01 A. The <(O-C-O) angle of the anchor is not affected by the binding to the substrate. The twist angle <(COOH-indolinium) between the plane of the anchor and the plane of indolinium was negligible for the isolated dyes. For the adsorbed dyes the twist angle is no longer negligible but still small, varying between 1.5° and 7.0°, with the largest value for OMCD1. The small twist allows for structural alignment, which favours the delocalization of the π electrons.

Looking more closely at the π bridge, the backbone of the three C atoms connecting the indolinium and benzoindolinium groups is more widely spread in the case of adsorbed dyes. The twist angle <(ind-benzoind) departs from the values of the bare dyes (12.2° to 15.5°) to either 21.5° to 26.9° or 1.3°. The departure from co-planarity can hinder electron delocalization and the intramolecular charge transfer along OMCD1 and OMCD2.

The donor part of the dye is tied to the benzoindolinium moiety through a –C≡C– group, whose length is slightly but systematically longer for the adsorbed dyes. The twist angles <(benzoind-ring), between the planes of the two groups, display significant differences, particularly for OMCD2, for which the two planes are almost perpendicular. Finally, the distances between the I atom and the N atom on the indolinium group are systematically smaller, by up to 0.68 Å. Due to the charge redistribution, I is pulled closer to the dye.

**Figure 7:**
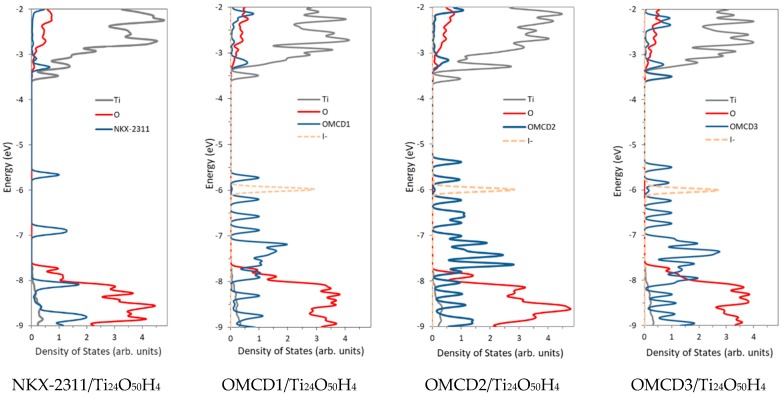
Density of states of the dye-oxide systems calculated in ethanol at the DFT/B3LYP/DZVP level. The contributions of the various atoms are: Ti—grey, O—red, dye molecules—blue, I-—orange. Energy levels were convoluted with Gaussian distributions with full width at half maximum 0.1 eV.

The density of states plots for the adsorbed dyes are represented in Figure 7. For all systems, the valence band is dominated by the *p* atomic orbitals of oxygen in the TiO_2_ nanocluster with significant contributions from the dye. In contrast, the conduction band has dominant contributions from the *d* atomic orbitals of Ti.

**Figure 8:**
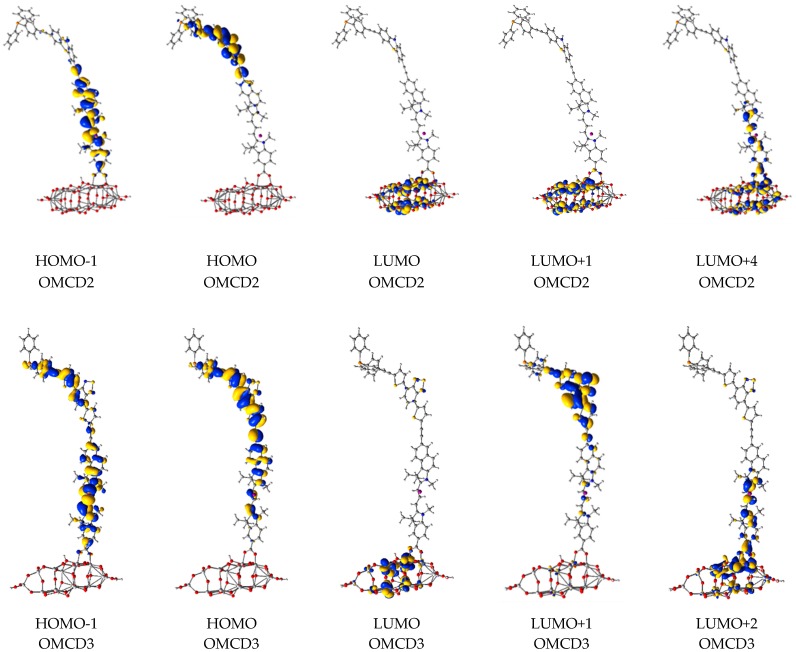
Isodensity surfaces (0.03 e/bohr^3^) of the key molecular orbitals of the OMCD2/Ti_24_O_50_H_4_ and OMCD3/Ti_24_O_50_H_4_ complex systems, calculated by DFT at B3LYP/DZVP level in ethanol.

The most relevant contributions of the adsorbed molecules are in the gap, where the HOMO of the complex systems is located, and in the conduction band, where the excited state of the dye lie. The ground state of each dye has negligible contribution from the cluster, whereas the excited state has mixed character, as it can be seen in Figure 8, which displays the electron density of the most important states for OMCD2 and OMCD3.

As mentioned in the previous section, a simple analysis of the energy level alignment can indicate whether the charge injection from the dye to the semiconducting oxide as well as of the regeneration of the dye by charge transfer from the electrolyte are possible [38,40]. The density of states plots reveal that the excited states of the dye lie within the CB of the semiconducting oxide, as expected, allowing for the transfer of the photoelectron from the dye to the substrate. Similarly, the dye regeneration through electron transfer from the electrolyte to the ground state of the dye is allowed for all dyes, as their HOMOs lie below the redox level of the I_3_^−^/I^−^ electrolyte, of −5.040 eV [53].

In the gap, under the ground state, there are several other states with π character, which are important for the optical spectra of the dyes. In particular, it is useful to examine the charge density of HOMO-1 and HOMO states (Figure 8) to understand the electron injection from the dye into the semiconductor. The charge of the HOMO-1 and HOMO is localized mostly on the donor unit and on the π bridge of the dye, whereas for the LUMO (the conduction band edge of TiO_2_) it is delocalized over the cluster.

Worth pointing out here are the charge distributions of the HOMO and HOMO-1 of the adsorbed OMCD2 dye. The two states are 0.47 eV apart, and while the charge on the HOMO is well localized on the donor, the charge on the HOMO-1 is delocalized over the bridge and the acceptor.

Peculiar for the OMCD3 dye is that the first excited state of the dye, LUMO+1 (located at −3.484 eV), has the charge localized on the donor, providing an example of a dye holding the charge instead of pushing it to the substrate. In contrast, the next excited state, LUMO+2 (at −3.218 eV), has the electron density distributed mostly on the acceptor and on the oxide, illustrating how the charge can be injected into the semiconductor. The two states are 0.266 eV apart.

For the other adsorbed dyes, the first excited state display push→pull effects compared to the ground state, as it can be seen from the electron density plots included in the Supplementary Materials. To have a quantitative measure of the push→pull effects we calculated the contributions of the various building units of the complex dye-substrate systems to the electron density of the key molecular orbitals. Examining the distribution of charge reported in Table 5, we note that the HOMOs have negligible contribution from the TiO_2_ cluster. Extremely small contributions from the dyes are present in the LUMOs, which represent the CB edge of the semiconducting oxide.

Worth examining in more detail are the excited states of the adsorbed dyes (LUMO+1 for OMCD1. LUMO+4 for OMCD2, and LUMO+2 for OMCD3), for which 75% or more of the charge is already transferred to the cluster. Of all dyes OMCD2 transfers most charge to the substrate, 80%. Moreover, OMCD2 displays the most powerful push→pull effect, as 99% of the charge of its HOMO (located mostly on the donating unit) is relocated in the LUMO+4 on the cluster and the acceptor unit. In contrast, OMCD1 starts in the HOMO with a large charge on the acceptor and only 20% is relocated from the donor and bridge. In the case of OMCD3, if we consider LUMO+2 instead of the first excited state of the adsorbed molecule (which holds the charge captive on the dye), we count 73% of the charge as being relocated from the donor and bridge (2% remaining on the bridge).

**Figure 9:**
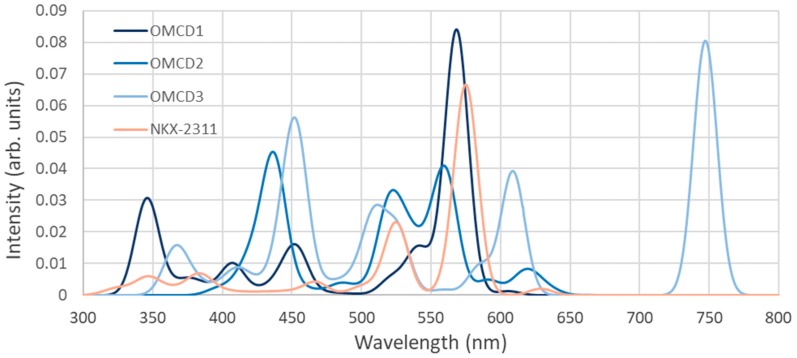
Theoretical UV-Vis spectra by TD-DFT calculation at B3LYP/DZVP level for the dyes on the Ti_24_O_50_H_4_ cluster in ethanol. The spectral lines were convoluted with Gaussian distributions of 1000 cm^−1^ linewidth at half maximum.

To study the matching of the UV-Vis absorption spectrum with the solar irradiation spectrum we determined theoretically the optical properties of the adsorbed dyes by TD-DFT calculations in ethanol solvent for all four dyes (see Figure 9). Compared to the absorption of the isolated dyes, the spectra of the adsorbed dyes are significantly modified.

For OMCD1, the main absorption bands are red-shifted, the highest wavelength (lowest energy) peak at 538 nm moving at 569 nm. Similarly, the other two peaks in the visible range moved from 435 nm and 399 nm to 540 and 454 nm. The first band mixes contributions from HOMO→LUMO+1 and HOMO→LUMO+2 transitions, and has an intensity about ten times stronger than the next bands. The second and third bands connect states below the HOMO with the first two excited states. The following bands are in the UV, connecting deeper states of the dye to higher states in the CB.

In the case of OMCD2, the shift of the main band is unusual, in the opposite direction, from 597 nm for the isolated dye to 562 nm for the adsorbed dye. The reason is that the first transition connects not the ground state but HOMO-1 to LUMO+1 and LUMO+4. To understand the spectra it is useful to return to Figure 8 and compare the charge distributions of the HOMO and HOMO-1 states.

As the intensity of the optical transition depends on the matrix element of the electric dipole moment between the two states, the overlap between the MOs involved may give a first clue regarding the probability of that transition. In the case of HOMO of OMCD2, the localization of charge far away from the anchor, with no contribution on the cluster leads to a negligible overlap with the excited states. In contrast, in the case of HOMO-1 the electron density gives a significant overlap with the excited states.

The other bands of OMCD2 are located in green, at 551, 534 and 520 nm, with contributions from HOMO-1 and HOMO-4 suffering transitions to LUMO+4 and LUMO+5 states. The transition in blue, at 437 nm, connects HOMO with states deeper into the CB (LUMO+43 and LUMO+44).

Finally, for OMCD3 the spectrum is again red-shifted, with strongest band at 747 nm, due to a HOMO→LUMO+1 transition. Other bands are in orange, at 609 nm (HOMO→LUMO+2), in yellow, at 585 nm (HOMO-1→LUMO+1), in green, at 526 nm (HOMO-1→LUMO+2) and 509 nm (HOMO-1→LUMO+3), and in blue, at 453 (HOMO→LUMO+29).

For comparison, the peaks for NKX-2311 are red-shifted to the yellow-green region, at 575 and 525 nm, in blue, at 467 nm and UV, at 384 nm 347 nm. Details regarding the transitions, particularly the oscillator strength and the major contributions, can be found in the Supplementary Materials.

Here again, to compare the matching of the UV-Vis absorption bands of the adsorbed dyes with the solar spectrum we calculated the area under the curves obtained multiplying the calculated spectra of the dyes and the standard global total spectral irradiance AM1.5 G [88]. The results are displayed in Table 2, and the resulting curves are provided in the Supplementary Materials. It can be seen that the best matching is obtained for OMCD3.

## 4. Discussion

### 4.1. Structure-Property Correlations—The Influence of the Molecular Building Blocks

In this section, we attempt a structure-property correlation, to better understand the roles of the various molecular building blocks of the three dyes. As stated in the introduction, the dyes were designed to serve as TiO_2_ sensitizers in DSSCs. Therefore, the building blocks were chosen to fulfil the requirements for such sensitizers, starting from the binding to the substrate and continuing with the push→pull effects and the energy level alignment and ending with the light harvesting [10,20].

To start with the common features, the existence of the –COOH group as an anchor fulfils for all three dyes the requirement for adsorption onto the TiO_2_ anatase surface. Our DFT calculations revealed the expected bidentate bridging configuration, with the proton bound to a nearby surface oxygen atom. The geometry of the binding is, overall, similar for all dyes, but small details differ. For instance, the <(O-C-O)*_a_* angles are almost identical, whereas the Ti–O distances show small differences. Some of the differences may reflect the tendency to take an upright position with respect to the cluster surface, as indicated by the dihedral angles between the indolinium plane and the plane of the three neighbour Ti atoms on the cluster surface, which show some variations around 80°.

The alkyl groups were introduced to improve solubility in organic solvents and to hinder dye aggregation during immobilization on the semiconductor. The distributions of charge shown in Figure 4 and Figure 8 indicate that they also play a role in the localization of charge closer to the backbone, as they stop the π-symmetry.

The triphenyl phosphonine group terminates all three dyes via ethynyl bonds. The charge distribution plots for the ground states indicate that only one of the phenyl rings participates in the charge delocalization due to the braking of the *π* symmetry. The *π* conjugation ends at the phosphorous atom in *sp*^3^ hybridization, and does not extend to the remaining two phenyl rings. Moreover, in all three cases, the terminating phenyl ring that holds the charge in the ground state passes it away through the *π*-bridge in the excited state. Therefore, the triphenyl phosphonine is efficient in both hindering dye aggregation, keeping dye molecules apart, but also in donating the charge in the excited state.

All dyes have a structure consisting of the typical D−π−A succession [10,24,25,26] of an electron donating group, a π-bridge and a charge acceptor ending in a carboxyl anchor. The dyes have, to a large extent, the conjugation that allows the π-symmetry needed for intramolecular charge transfer and electron injection into the substrate.

The electron donor part of all three dyes has different blocks to connect to the *π*-bridge, namely the ethynyl bonds, the phenothiazine, or the benzothiadiazole in between two thiophene groups. When designing the dyes, the expectation was that their absorption spectra would be red-shifted by lengthening the conjugated backbone. Indeed, the extension of the π conjugation in the present series of molecules resulted in red-shifting the first absorption peak from 538 nm to 597 nm or 653 nm in the case of OMCD1, OMCD2, or OMCD3, respectively, increasing the overlap of the UV-Vis spectrum with the solar one.

The different building blocks led to small structural differences occurred, for instance in the torsion angle between the anchor and the indolinium group, <(COOH-ind), is 7°, small but still significant, for OMCD1, compared to the other dyes. Similarly, the twist angle between the acceptor and the bridge, <(ind-benzoind), varies among the dyes again OMCD1 displaying the largest departure from planarity. These small twists may hinder the π-conjugation and the electron delocalization, which may affect the intramolecular charge transfer and the injection into the oxide.

The donating parts of the dyes have different behaviour in terms of pushing the charge away in the excited state. A depletion of electronic density occurs on the phenothiazine block (of OMCD2) during the transition from the ground to the excited state, whereas the benzothiadiazole and the surrounding thiophene groups (of OMCD3) retain a significant part of the charge, hindering the push→pull effects (Table 2 and Table 4).

The *π*-bridge and electron acceptor part are identical for all dyes. However, the tendency of charge injection for the benzo[e]indolinium and indolinium groups is clearly affected by the donor parts. In particular, as mentioned above, the tendency of the benzothiadiazole group to retain the charge seems more powerful than the possibility of the bridging blocks to pass it. In addition, these trends, observed for the isolated dyes, are also confirmed for the dyes adsorbed on substrate.

### 4.2. Comparative Analysis of the Oligomethine Dyes

In this section, we make a comparison of the three oligomethine dyes as candidates for TiO_2_ sensitizers, taking as reference the coumarin-based dye. To compare the dyes, we start from the criteria that have to be met by a dye to be an efficient TiO_2_ sensitizer in DSSCs, as reviewed in the introduction [10,20].

First, the requirement for adsorption onto the TiO_2_ anatase surface is fulfilled equally well by all dyes. We note that the holistic approach that takes into consideration the dye and the substrate together has the advantage of illustrating how the charge is distributed and injected into the oxide. In case of orthogonal orbitals between the dye and the substrate the charge transfer is hindered [89], an example being offered by dyes with *π*-symmetry ending in carboxyl anchors and a semiconductor such as ZnO, with the same band gap as TiO_2_ [5] but which has the conduction band dominated by the *s* atomic orbitals of the metal. In contrast, the conduction band of TiO_2_ has *d* atomic orbitals involved, as shown in our density of states calculations (Figure 7), allowing for strong overlap with the *π* orbital of the dye [89]. DFT calculations of the dye-substrate system can reveal the existence of such bottlenecks for the photoelectron injection.

Second, although all dyes have a D−π−A structure and the π-conjugation that allows the charge delocalization, some structural differences cause significant property variances, as small twists may hinder the π-symmetry, which in turn may affect the intramolecular charge transfer. For instance, the structural effect the cluster has on the <(benzoind-ring) dihedral angle of the adsorbed OMCD2 dye, for which the planes become almost perpendicular, is reflected in the electron density of the key states. The disruption of the π-symmetry tends to separate the charge on either side, which, surprisingly, has a positive effect on pushing the charge towards the TiO_2_ substrate.

Overall, the push→pull effects are consistent for the isolated and adsorbed dyes, as reported in Table 2 and Table 5. Of all dyes, OMCD2 displays the most powerful push→pull effect in both isolated and adsorbed situations. In contrast, OMCD3, tends to keep the charge in the excited state, only higher excited states allowing push→pull behavior. Our results indicate that OMCD2 and OMCD1 (and even OMCD3 if we count the transitions to the LUMO+2 state) outperform NKX-2311 in terms of intramolecular charge transfer.

Third, we analyzed the energy level alignment between the dye, the oxide and the electrolyte, as they influence the electron injection and the dye regeneration. The charge injection is possible if the exited state of the dye lies above the conduction band edge, whereas the dye regeneration is feasible if the redox level of the electrolyte lies higher than the ground state of the dye. Our study showed consistent results for both the isolated and the adsorbed dyes, as revealed by the energy level diagram in Figure 3 and the densities of states displayed in Figure 7. At a qualitative level, all four dyes have the proper energy level alignment that allows both the injection of the photoelectron into the oxide and the regeneration of the dye by the electrolyte.

Looking more closely at the charge injection, the driving forces determined for the bare dyes are comparable, being largest, by a thin margin, for NKX-2311 (0.47 eV). OMCD2 comes second with 0.44 eV, OMCD1 third with 0.43 eV, whereas for OMCD3 it is as low as 0.28 eV. The values of the driving force for the adsorbed dyes can be measured from the edge of the conduction band, leading to values of 0.483, 0.574, 0.700 and 0.567 eV, for NKX-2311, OMCD1, OMCD2 and OMCD3, respectively. Calculating the difference in energy between the peaks in the density of states for the excited state of the adsorbed dye and for the first state in the conduction band of TiO_2_, results in smaller values, of 0.189, 0.280, 0.406 and 0.273 eV, respectively. Either way, OMCD1 and OMCD2 outperform NKX- 2311. If we take into account the LUMO+2 state, even OMCD3 has a larger driving force than the coumarin-based dye.

We believe that the more trustworthy analysis is the one involving the dyes adsorbed onto the TiO_2_ nanocluster, which takes into account the interactions, such as the interface effects caused by the binding of the dye, as well as the energy minimization by means of structural distortions. Moreover, the analysis of the density of states for the dye-cluster system may provide additional information regarding the existence at the energy of the excited state of the dye of another state of similar symmetry in the conduction band of the semiconducting oxide, in case that could facilitate the injection of the photoelectron.

Fourth, we investigated the light-harvesting ability of the dyes by determining the matching of their absorption spectra with the solar emission spectrum. The electronic absorption spectra presented in Figure 5 and Figure 9 and the calculation of the matching shown in Table 3, in particular, provide a clear comparison of the four dyes. All three oligomethine dyes outperform the coumarin-based dye in terms of matching the solar spectrum, OMCD3 showing the best light harvesting, followed by OMCD2 and OMCD1.

We note that while the results obtained for the bare dyes are consistent with those obtained for the dye-oxide system, quantitatively there are some significant differences between the two approaches. Given that most molecular orbitals of the complex system have a mixt character, transitions that are forbidden for the isolated dye may become allowed when the dye is adsorbed on the nanocluster. Consequently, the absorption spectra of the adsorbed dyes are more likely to describe the real system than the spectra of the dyes alone.

In summary, the three dyes reported here meet to various extents the basic requirements for TiO_2_ sensitizers. OMCD3 likely has the largest light harvesting efficiency but may suffer in terms of electron transfer and injection into the substrate. At the other end, OMCD1 has the weakest absorption and an intermediate tendency for push→pull effects. OMCD2 seems to have a potential for significant light harvesting and electron injection and the strongest intramolecular charge transfer properties. Under these circumstances, as it is impossible to single out a molecule that has best performance in all aspects, it is difficult to predict the dye likely to cause the highest photovoltaic conversion efficiency. Given the positive comparison with the reference dye, NKX-2311 for which the experimental efficiency was 5.6% [43,44], we think that the three dyes reported have the potential to overcome such performance. We venture to propose OMCD2 as the strongest candidate of the three as TiO_2_ sensitizer.

## 5. Conclusions

We reported the synthesis of three oligomethine cyanine-based dyes as candidates for DSSC sensitizers, emphasizing their structural, electronic and optical properties, to evaluate their practical applicability. The goal of the present study was to determine whether the three dyes are good candidates for photovoltaic applications and to analyze the effect of the different substitutions on the electronic and optical properties of these sensitizers. A secondary goal was to better understand, through structure-property correlations, the influence of the various molecular building blocks on dye performance as TiO_2_ sensitizer.

To study their structural, electronic and optical properties, we performed DFT and TD-DFT calculations on isolated as well as adsorbed dyes and analyzed whether they fulfil the criteria for sensitizing TiO_2_. While the carboxyl group allows, as anchor, for bidentate bridging bonding to the substrate, bidentate bridging, the alkyl groups hinder dye aggregation and play a role in the localization of charge closer to the backbone, as they stop the π-symmetry. The triphenyl phosphonine group that terminates all three dyes play a role in impeding dye aggregation and in the charge transfer. Triphenyl phosphonine keeps dye molecules apart and breaks the π-symmetry allowing only one of the three phenyl rings to be involved in intramolecular electron transfer. Indolinium and benzo[e]indolinium are efficient π-bridge constituents or acceptors, whereas phenothiazine plays the role of an electron donor. In contrast, benzothiadiazole retains a significant part of the charge, hindering the transfer.

The comparison of the three dyes, keeping the coumarin-based NKX-2311 dye as reference, indicated that they all meet, to various degrees, the requirements of anchoring, light harvesting, intramolecular charge transfer and electron injection into the substrate, dye regeneration, etc. Moreover, they seem to outperform the 5.6% efficiency reference dye, which raises hopes for the applicability of the three oligomethine dyes. While OMCD3 has the best matching with the solar spectrum but the weakest electron injection, OMCD1 has the weakest absorption and an intermediate propensity for push→pull effects. OMCD2 may be the strongest candidate as TiO_2_ sensitizer, with significant light harvesting and the most facile charge transfer.

Finally, the comparison of the results obtained for the free dye with those obtained for the combined dye-oxide system showed the superiority of the more holistic approach for studying anchoring, energy level alignment and charge injection as well as dye regeneration, charge transfer, optical spectra, etc. DFT calculations have strong explanatory and predictive power, and can be an efficient tool to assist in the optimization of dye-sensitized solar cells.

## Figures and Tables

**Table 1:** The structural parameters of the dyes, obtained after geometry optimization by DFT at B3LYP/DZVP level in ethanol. Geometrical parameters describing the dyes: bond lengths (in Å), the torsion angle of the carboxyl group relative to the indolinium plane, and the dihedral angle defined by the indolinium plane and the plane of the three neighbor Ti atoms on the cluster surface (both in deg).

Parameter	OMCD1	OMCD2	OMCD3	OMCD1	OMCD2	OMCD3
				on Ti_24_O_50_H_4_		
*r*(C*_a_*-C*_i_*)	1.483	1.483	1.483	1.472	1.474	1.472
<(O-C-O)*_a_*	121.8	121.8	121.8	121.5	121.6	121.6
<(COOH-ind)	0.2	0.0	0.5	7.0	1.5	2.3
*r1*(C*_i_*-C*_bi_*)	1.394	1.394	1.394	1.397	1.398	1.396
*r2*(C*_i_*-C*_bi_*)	1.406	1.407	1.407	1.414	1.414	1.416
*r3*(C*_i_*-C*_bi_*)	1.397	1.398	1.397	1.403	1.404	1.402
*r4*(C*_i_*-C*_bi_*)	1.403	1.404	1.403	1.409	1.409	1.409
*r*(C*_i_*-C*_bi_*)	5.066	5.066	5.067	5.071	5.076	5.093
<(ind-benzoind)	15.5	14.7	12.2	26.9	21.5	1.3
*r1*(C*_bi_*-C*_r_*)	1.428	1.429	1.425	1.432	1.436	1.428
*r2*(C*_bi_*-C*_r_*)	1.221	1.221	1.223	1.229	1.229	1.231
*r3*(C*_bi_*-C*_r_*)	1.429	1.428	1.408	1.433	1.435	1.406
*r*(C*_bi_*-C*_r_*)	4.078	4.076	4.055	4.094	4.099	4.065
<(benzoind-ring)	2.5	21.1	13.6	11.5	88.1	4.5
*r*(I-N*_i_*)	4.966	5.141	5.034	4.421	4.465	5.020

**Table 2:** Contributions of the donor, π-bridge and acceptor groups to the electron density of the main molecular orbitals of de dyes, calculated at the DFT/B3LYP/DZVP level. For the oligomethine cyanine dyes the various units consist of the carboxyl anchor and the indolinium group, for the acceptor, the benzoindolinium group for the bridge and the various terminations for the donor.

Dye	MO	Donor unit (%)	π-bridge unit (%)	Acceptor unit (%)
NKX-2311	LUMO	40	38	22
	HOMO	78	10	12
OMCD1	LUMO	1	1	98
	HOMO	11	8	80
OMCD2	LUMO	2	38	60
	HOMO	85	11	4
OMCD3	LUMO+1	0	16	84
	LUMO	2	81	17
	HOMO	7	67	26

**Table 3:** Spectral matching coefficient, between the calculated spectra of the dyes and the standard global total spectral irradiance AM1.5 G [88], based on Equation (8), for the isolated dye and the dye adsorbed on the TiO_2_ nanocluster, for wavelengths between 300 and 900 nm.

Dye	OMCD1	OMCD2	OMCD3	NKX-2311
Isolated dye	4.107	5.588	7.112	2.591
Adsorbed dye	4.746	5.307	7.837	3.552

**Table 4:** The structural parameters of the model system consisting of the Ti_24_O_50_H_4_ clusters with an adsorbed dye molecule, and of the bare cluster, obtained after geometry optimization by DFT/B3LYP/LANL2DZ in ethanol. Geometrical parameters describing the adsorption modes: bond lengths (in Å), the torsion angle of the carboxyl group relative to the indolinium plane, and the dihedral angle defined by the indolinium plane and the plane of the three neighbor Ti atoms on the cluster surface (both in deg).

Parameter	OMCD1/Ti_24_O_50_H_4_	OMCD2/Ti_24_O_50_H_4_	OMCD3/Ti_24_O_50_H_4_	NKX-2311/Ti_24_O_50_H_4_
*r*(Ti-O)	1.876	1.899	1.877	1.891
*σ_r_*(Ti-O)	0.104	0.115	0.106	0.116
*r*(O1-Ti)	2.082	2.067	2.085	2.094
*r*(O2-Ti)	1.942	1.994	1.941	1.987
<(O-C-C-C)	4.8	2.0	2.7	3.7
<(Phenyl;3Ti)	75.6	76.9	86.4	86.0

**Table 5:** Contributions of the donor, π-bridge, acceptor units and the Ti_24_O_50_H_4_ nanocluster to the electron density of the main molecular orbitals of the adsorbed dyes, calculated at the DFT/B3LYP/DZVP level.

Dye	MO	Donor (%)	Bridge (%)	Acceptor (%)	Cluster (%)
NKX-2311 /Ti_24_O_50_H_4_	LUMO+1	9	10	6	75
	LUMO	0	1	1	98
	HOMO	77	11	12	0
OMCD1 /Ti_24_O_50_H_4_	LUMO+1	0	0	24	76
	LUMO	0	0	1	99
	HOMO	12	8	80	0
OMCD2 /Ti_24_O_50_H_4_	LUMO+4	0	0	20	80
	LUMO	0	0	1	99
	HOMO	96	3	1	0
	HOMO-1	8	57	35	0
OMCD3 /Ti_24_O_50_H_4_	LUMO+2	0	2	23	75
	LUMO+1	2	87	7	4
	LUMO	0	3	1	96
	HOMO	6	69	25	0

## Supplementary Materials

The following are available online at https://www.mdpi.com/2079-4991/10/4/662/s1, Schemes S1–S3: synthesis schemes, Figures S1–S22: FTIR and NMR spectra of compounds synthesized, Table S1: Characterization data for intermediate compounds and oligomethine cyanine dyes OMCD1–3, Figure S23: Visible spectra of dyes OMCD1, OMCD2, and OMCD3 in ethanol, Figure S24 Simulated UV-Vis spectra of isolated dyes, Figure S25: Matching with the solar spectrum of the absorption spectra of isolated dyes, Table S2: Wavelength, oscillator strength and composition of electronic transitions, Table S3: Contributions of the building blocks to the electron density of the main molecular orbitals of the adsorbed dyes, Figure S26: Isodensity surfaces of the key molecular orbitals of adsorbed dyes, Figure S27: Simulated UV-Vis spectra of adsorbed dyes, Figure S28: Matching with the solar spectrum of the absorption spectra of adsorbed dyes, Table S4: Wavelength, oscillator strength and composition of electronic transitions.

## References

[1] Trancik J.E., Cross-Call D. (2013). Energy technologies evaluated against climate targets using a cost and carbon trade-off curve. Environ. Sci. Technol..

[2] Kavlak G., McNerney J., Trancik J.E. (2018). Evaluating the causes of cost reduction in photovoltaic modules. Energy Policy.

[3] Green M.A. (2019). Photovoltaic technology and visions for the future. Prog. Energy.

[4] O’Regan B., Grätzel M. (1991). A low-cost, high-efficiency solar cell based on dye-sensitized colloidal TiO2 films. Nature.

[5] Grätzel M. (2001). Photoelectrochemical cells. Nature.

[6] Gong J., Sumathy K., Qiao Q., Zhou Z. (2018). Review on dye-sensitized solar cells (DSSCs): Advanced techniques and research trends. Renew. Sustain. Energy Rev..

[7] Ahn K.S., Yoo S.J., Kang M.S., Lee J.W., Sung Y.E. (2007). Tandem dye-sensitized solar cell-powered electrochromic devices for the photovoltaic-powered smart window. J. Power Sources.

[8] Baetens R., Jelle B.P., Gustavsen A. (2010). Properties, requirements and possibilities of smart windows for dynamic daylight and solar energy control in buildings: A state-of-the-art review. Sol. Energy Mater. Sol. Cells.

[9] Freitag M., Teuscher J., Saygili Y., Zhang X., Giordano F., Liska P., Hua J., Zakeeruddin S.M., Moser J.-E., Grätzel M. (2017). Dye-sensitized solar cells for efficient power generation under ambient lighting. Nat. Photonics.

[10] Hagfeldt A., Boschloo G., Sun L., Kloo L., Pettersson H. (2010). Dye-Sensitized Solar Cells. Chem. Rev..

[11] Nazeeruddin M.K., Pechy P., Renouard T., Zakeeruddin S.M., Humphry-Baker R., Comte P., Liska P., Cevey L., Costa E., Shklover V. (2001). Engineering of efficient panchromatic sensitizers for nanocrystalline TiO(2)-based solar cells. J. Am. Chem. Soc..

[12] Nazeeruddin M.K., De Angelis F., Fantacci S., Selloni A., Viscardi G., Liska P., Ito S., Bessho T., Grätzel M. (2005). Combined Experimental and DFT-TDDFT Computational Study of Photoelectrochemical Cell Ruthenium Sensitizers. J. Am. Chem. Soc..

[13] Xie Y., Tang Y., Wu W., Wang Y., Liu J., Li X., Tian H., Zhu W.-H. (2015). Porphyrin Cosensitization for a Photovoltaic Efficiency of 11.5%: A Record for Non-Ruthenium Solar Cells Based on Iodine Electrolyte. J. Am. Chem. Soc..

[14] Yella A., Lee H.W., Tsao H.N., Yi C., Chandiran A.K., Nazeeruddin M.K., Diau E.W.G., Yeh C.Y., Zakeeruddin S.M., Grätzel M. (2011). Porphyrin-sensitized solar cells with cobalt (II/III)–based redox electrolyte exceed 12 percent efficiency. Science.

[15] Mathew S., Yella A., Gao P., Humphry-Baker R., Curchod B.F.E., Ashari-Astani N., Tavernelli I., Rothlisberger U., Nazeeruddin M.K., Grätzel M. (2014). Dye-sensitized solar cells with 13% efficiency achieved through the molecular engineering of porphyrin sensitizers. Nat. Chem..

[16] Kakiage K., Aoyama Y., Yano T., Oya K., Kyomen T., Hanaya M. (2015). Fabrication of a high-performance dye-sensitized solar cell with 12.8% conversion efficiency using organic silyl-anchor dyes. Chem. Commun..

[17] Yao Z., Wu H., Li Y., Wang J., Zhang J., Zhang M., Guo Y., Wang P. (2015). Dithienopicenocarbazole as the kernel module of low-energy-gap organic dyes for efficient conversion of sunlight to electricity. Energy Environ. Sci..

[18] Eom Y.K., Kang S.H., Choi I.T., Yoo Y., Kim J., Kim H.K. (2017). Significant light absorption enhancement by a single heterocyclic unit change in the p-bridge moiety from thieno[3,2-b]benzothiophene to thieno[3,2-b]indole for high performance dye-sensitized and tandem solar cells. J. Mater. Chem..

[19] Zhang L., Yang X., Wang W., Gurzadyan G.G., Li J., Li X., An J., Yu Z., Wang H., Cai B. (2019). 13.6% efficient organic dye-sensitized solar cells by minimizing energy losses of the excited state. ACS Energy Lett..

[20] Hamann T.W., Jensen R.A., Martinson A.B.F., van Ryswyk H., Hupp J.T. (2008). Advancing beyond current generation dye-sensitized solar cells. Energy Environ. Sci..

[21] O’Regan B.C., Durrant J.R. (2009). Kinetic and energetic paradigms for dye-sensitized solar cells: Moving from the ideal to the real. Acc. Chem. Res..

[22] Snaith H.J. (2010). Estimating the Maximum Attainable Efficiency in Dye-Sensitized Solar Cells. Adv. Funct. Mater..

[23] Peter L.M. (2011). The Grätzel cell: Where next?. J. Phys. Chem. Lett..

[24] Mishra A., Fischer M.K., Bauerle P. (2009). Metal-free organic dyes for dye-sensitized solar cells: From structure: Property relationships to design rules. Angew. Chem. Int. Ed..

[25] Imahori H., Umeyama T., Ito S. (2009). Large pi-aromatic molecules as potential sensitizers for highly efficient dye-sensitized solar cells. Acc. Chem. Res..

[26] Clifford J.N., Martinez-Ferrero E., Viterisi A., Palomares E. (2011). Sensitizer molecular structure-device efficiency relationship in dye sensitized solar cells. Chem. Soc. Rev..

[27] Wu Y., Zhu W. (2013). Organic sensitizers from D-pi-A to D-A-pi-A: Effect of the internal electron-withdrawing units on molecular absorption, energy levels and photovoltaic performances. Chem. Soc. Rev..

[28] Sayama K., Hara K., Ohga Y., Shinpou A., Suga S., Arakawa H. (2001). Significant effects of the distance between the cyanine dye skeleton and the semiconductor surface on the photoelectrochemical properties of dye-sensitized porous semiconductor electrodes. New J. Chem..

[29] Matsui M., Hashimoto Y., Funabiki K., Jin J.Y., Yoshida T., Minoura H. (2005). Application of near-infrared absorbing heptamethine cyanine dyes as sensitizers for zinc oxide solar cell, Synth. Metals.

[30] Otsuka A., Funabiki K., Sugiyama N., Mase H., Yoshida T., Minoura H., Matsui M. (2008). Design and synthesis of near-infrared-active heptamethine-cyanine dyes to suppress aggregation in a dye-sensitized porous zinc oxide solar cell. Chem. Lett..

[31] Ono T., Yamaguchi T., Arakawa H. (2009). Study on dye sensitized solar cell using novel infrared dye. Sol. Energy Mater. Sol. Cells.

[32] Funabiki K., Mase H., Hibino A., Tanaka N., Mizuhata N., Sakuragi Y., Nakashima A., Yoshida T., Kubota Y., Matsui M. (2011). Synthesis of a novel heptamethine-cyanine dye for use in near-infrared active dye sensitized solar cells with porous zinc oxide prepared at low temperature. Energy Environ. Sci..

[33] McArthur E.A., Godbe J.M., Tice D.B., Weiss E.A. (2012). A study of the binding of cyanine dyes to colloidal quantum dots using spectral signatures of dye aggregation. J. Phys. Chem. C.

[34] Hohenberg P., Kohn W. (1964). Inhomogeneous electron gas. Phys. Rev..

[35] Kohn W., Sham L.J. (1965). Self-consistent equations including exchange and correlation effects. Phys. Rev..

[36] Parr R.G., Yang W. (1989). Density-Functional Theory of Atoms and Molecules.

[37] Casida M.E., Jamorski C., Casida K.C., Salahub D.R. (1998). Molecular excitation energies to high-lying bound states from time-dependent Density-Functional Response theory: Characterization and correlation of the time dependent local density approximation ionization threshold. J. Chem. Phys..

[38] De Angelis F., Fantacci S., Selloni A., Grätzel M., Nazeeruddin M.K. (2007). Influence of the sensitizer adsorption mode on the open-circuit potential of dye-sensitized solar cells. Nano Lett..

[39] De Angelis F., Fantacci S., Mosconi E., Nazeeruddin M.K., Grätzel M. (2010). First-Principles Modeling of the Adsorption Geometry and Electronic Structure of Ru(II) Dyes on Extended TiO2 Substrates for Dye-Sensitized Solar Cell Applications. J. Phys. Chem. C.

[40] Oprea C.I., Dumbrava A., Enache I., Lungu J., Georgescu A., Moscalu F., Oprea C., Gîrţu M.A. (2011). Role of energy level alignment in solar cells sensitized with a metal free organic dye: A combined experimental and theoretical approach. Phys. Status Solidi A.

[41] Oprea C.I., Dumbrava A., Enache I., Georgescu A., Gîrţu M.A. (2012). A combined experimental and theoretical study of natural betalain pigments used in dye-sensitized solar cells. J. Photochem. Photobiol. A Chem..

[42] Oprea C.I., Panait P., Cimpoesu F., Ferbinteanu M., Gîrţu M.A. (2013). Density Functional Theory (DFT) Study of Coumarin-based Dyes Adsorbed on TiO2 Nanoclusters—Applications to Dye-Sensitized Solar Cells. Materials.

[43] Hara K., Sayama K., Arakawa H., Ohga Y., Shinpo A., Suga S. (2001). A coumarin-derivative dye sensitized nanocrystalline TiO2 solar cell having a high solar-energy conversion efficiency up to 5.6%. Chem. Commun..

[44] Hara K., Sato T., Katoh R., Furube A., Ohga Y., Shinpo A., Suga S., Sayama K., Sugihara H., Arakawa H. (2003). Molecular design of coumarin dyes for efficient dye-sensitized solar cells. J. Phys. Chem. B.

[45] Pirotte G., Agarkar S., Xu B., Zhang J., Lutsen L., Vanderzande D., Yan H., Pollet P., Reynolds J.R., Maes W. (2017). Molecular weight tuning of low bandgap polymers by continuous flow chemistry: Increasing the applicability of PffBT4T for organic photovoltaics. J. Mater. Chem. A.

[46] Wu Y., Guo H., Shao J., Zhang X., Ji S., Zhao J. (2011). Synthesis of Ethynylated Phenothiazine Based Fluorescent Boronic Acid Probes. J. Fluoresc..

[47] Miyaura N., Suzuki A. (1995). Palladium-Catalyzed Cross-Coupling Reactions of Organoboron Compounds. Chem. Rev..

[48] Stille J.K. (1986). The Palladium-Catalyzed Cross-Coupling Reactions of Organotin Reagents with Organic Electrophiles [New Synthetic Methods (58)]. Angew. Chem. Int. Ed. Engl..

[49] Becke A.D. (1993). Density-functional thermochemistry. III. The role of exact exchange. J. Chem. Phys..

[50] Lee C., Yang W., Parr R.G. (1988). Development of the colle-salvetti correlation-energy formula into a functional of the electron density. Phys. Rev. B.

[51] Godbout N., Salahub D.R., Andzelm J., Wimmer E. (1992). Optimization of Gaussian-type basis sets for local spin density functional calculations. Part I. Boron through neon, optimization technique and validation. Can. J. Chem..

[52] Persson P., Lundqvist M.J. (2005). Calculated Structural and Electronic Interactions of the Ruthenium Dye N3 with a Titanium Dioxide Nanocrystal. J. Phys. Chem. B.

[53] De Angelis F., Fantacci S., Selloni A. (2008). Alignment of the dye’s molecular levels with the TiO2 band edges in dye-sensitized solar cells: A DFT-TDDFT study. Nanotechnology.

[54] Oprea C.I., Gîrțu M.A. (2019). Structure and Electronic Properties of TiO2 Nanoclusters and Dye–Nanocluster Systems Appropriate to Model Hybrid Photovoltaic or Photocatalytic Applications. Nanomaterials.

[55] Oprea C.I., Panait P., AbdelAal R.M., Gîrțu M.A. DFT Calculations of Structure and Optical Properties in Wide Band-Gap Semiconductor Clusters for Dye-Sensitized Solar Cells. Proceedings of the IEEE Proceedings International Semiconductor Conference (CAS).

[56] Hay P.J., Wadt W.R. (1985). Ab initio effective core potentials for molecular calculations. Potentials for K to Au including the outermost core orbitals. J. Chem. Phys..

[57] Barone V., Cossi M. (1998). Quantum calculation of molecular energies and energy gradients in solution by a conductor solvent model. J. Phys. Chem. A.

[58] Tomasi J., Mennucci B., Cammi R. (2005). Quantum mechanical continuum solvation models. Chem. Rev..

[59] Frisch M.J., Trucks G.W., Schlegel H.B., Scuseria G.E., Robb M.A., Cheeseman J.R., Scalmani G., Barone V., Petersson G.A., Nakatsuji H. (2016). Gaussian 09, Revision A.02.

[60] Peter J.M. (2007). Dye-sensitized nanocrystalline solar cells. Phys. Chem. Chem. Phys..

[61] Ferber J., Stangl R., Luther J. (1998). An electrical model of the dye-sensitized solar cell. Sol. Energy Mater Sol. Cells.

[62] Marinado T., Nonomura K., Nissfolk J., Karlsson M.K., Hagberg D.P., Sun L., Sun L., Hagfekdt A. (2010). How the nature of triphenylamine-polyene dyes in dye-sensitized solar cells affects the open-circuit voltage and electron lifetimes. Langmuir.

[63] Katoh R., Furube A., Barzykin A.V., Arakawa H., Tachiya M. (2004). Kinetics mechanism of electron injection and charge recombination in dye-sensitized nanocrystalline semiconductors. Coord. Chem. Rev..

[64] Rühle S., Greenshtein M., Chen S.G., Merson A., Pizem H., Sukenik C.S., Cahen D., Zaban A. (2005). Molecular adjustment of the electronic properties of nanoporous electrodes in dye-sensitized solar cells. J. Phys. Chem. B.

[65] Zhang C.R., Liu Z.J., Chen Y.H., Chen H.S., Wu Y.Z., Feng W., Wang D.B. (2010). DFT and TD-DFT study on structure and properties of organic dye sensitizer TA-St-CA. Curr. Appl. Phys..

[66] Sang-aroon W., Kunmuak K., Tontapha S., Chaiamornnugool P., Saekow S., Amornkitbamrung V. (2014). Theoretical insight into electronic and photoelectrochemical 3 properties of orcein dyes relevant to dye-sensitized solar cells. Monatsh. Chem..

[67] Hasanein A.A., Elmarassi Y.R., Kassem E.N. (2016). TD-DFT investigation of D-π-A organic dyes with thiophene moieties as π-spacers for use as sensitizers in DSSCs. J. Mol. Model..

[68] Liang G., Yuan Y., Wang D., Zhong Z., Xu J. (2014). Tuning the electronic structures and related properties of phenothiazine-based donor-π-acceptor dyes for dye-sensitized solar cells: A theoretical study. Monatsh. Chem..

[69] Fitri A., Benjelloun A.T., Benzakour M., Mcharfi M., Hamidi M., Bouachrine M. (2014). Theoretical investigation of new thiazolothiazole-based D-π-A organic dyes for efficient dye-sensitized solar cell. Spectrochim. Acta A.

[70] Arkan F., Izadyar M., Nakhaeipour A. (2015). A quantum chemistry study on the performance of porphyrin-based solar cell sensitisers; Zinc and anchor group position effects. Mol. Phys..

[71] Mohr T., Aroulmoji Z., Ravindran R.S., Muller M., Ranjitha S., Rajaranjan G., Anbarasan P.M. (2015). DFT and TD-DFT study on geometries, electronic structures and electronic absorption of some metal free dye sensitizers for dye sensitized solar cells. Spectrochim. Acta A.

[72] Mahmood A., Tahir M.H., Irfan A., Al-Sehemi A.G., Al-Assiri M.S. (2015). Heterocyclic azo dyes for dye sensitized solar cells: A quantum chemical study. Comput. Theor. Chem..

[73] Ren X.-F., Kang G.-J., He Q.-Q. (2016). Triphenylamine-based indoline derivatives for dye-sensitized solar cells: A density functional theory investigation. J. Mol. Model..

[74] Li Y., Mi L., Wang H., Li Y., Liang J. (2019). Design, Electron Transfer Process, and Opto-Electronic Property of Solar Cell Using Triphenylamine-Based D-π-A Architectures. Materials.

[75] Matthews D., Infelta P., Grätzel M. (1996). Calculation of the photocurrent-potential characteristic for regenerative, sensitized semiconductor electrodes. Sol. Energy Mater Sol. Cells.

[76] Pazoki M., Cappel U.B., Johansson E.M.J., Hagfeldt A., Boschloo G. (2017). Characterization techniques for dye-sensitized solar cells. Energy Environ. Sci..

[77] Nazeeruddin M.K., Kay A., Rodicio I., Humphry-Baker R., Mueller E., Liska P., Vlachopoulos N., Graetzel M. (1993). Conversion of light to electricity by cis-X2bis (2, 2′-bipyridyl-4, 4′-dicarboxylate) ruthenium (II) charge-transfer sensitizers (X= Cl-, Br-, I-, CN-, and SCN-) on nanocrystalline titanium dioxide electrodes. J. Am. Chem. Soc..

[78] Xie X., Liu Z.-H., Bai F.-Q., Zhang H.-X. (2019). Performance Regulation of Thieno[3,2-b]benzothiophene p-Spacer-Based D-p-A Organic Dyes for Dye-Sensitized Solar Cell Applications: Insights From Computational Study. Front. Chem..

[79] National Renewable Energy Laboratory American Society for Testing and Materials (ASTM), ASTM G-173-03 (2012) Standard Tables for Reference Solar Spectral Irradiances: Direct Normal and Hemispherical on 37° Tilted Surfaces. https://www.nrel.gov/grid/solar-resource/spectra-am1.5.html.

[80] He L.J., Wei W., Chen J., Jia R., Wang J., Zhang H.X. (2017). The effect of D–[D e–π–A] n (n= 1, 2, 3) type dyes on the overall performance of DSSCs: A theoretical investigation. J. Mater. Chem. C.

[81] Shi X., Yang Y., Wang L., Li Y. (2019). Introducing Asymmetry Induced by Benzene Substitution in a Rigid Fused π Spacer of D−π−A-Type Solar Cells: A Computational Investigation. J. Phys. Chem. C.

[82] Zhao D., Lu Q., Su R., Li Y., Zhao M. (2019). Light Harvesting and Optical-Electronic Properties of Two Quercitin and Rutin Natural Dyes. Appl. Sci..

[83] Ma W., Jiao Y., Meng S. (2014). Predicting Energy Conversion Efficiency of Dye Solar Cells from First Principles. J. Phys. Chem. C.

[84] Ren P., Sun C., Shi Y., Song P., Yang Y., Li Y. (2019). Global performance evaluation of solar cells using two models: From charge-transfer and recombination mechanisms to photoelectric properties. J. Mater. Chem. C.

[85] Scott A.P., Radom L. (1996). Harmonic vibrational frequencies: An evaluation of Hartree−Fock, Møller−Plesset, quadratic configuration interaction, density functional theory, and semiempirical scale factors. J. Phys. Chem..

[86] Anderson N.A., Lian T. (2004). Ultrafast electron injection from metal polypyridyl complexes to metal-oxide nanocrystalline thin films. Coord. Chem. Rev..

[87] Zhang X., Zhang J.-J., Xia Y.-Y. (2008). Molecular design of coumarin dyes with high efficiency in dye- sensitized solar cells. J. Photochem. Photobiol. A Chem..

[88] Martsinovich N., Troisi A. (2011). High-throughput computational screening of chromophores for dye-sensitized solar cells. J. Phys. Chem. C.

[89] Anderson S., Constable E.C., Dare-Edwards M.P., Goodenough J.B., Hamnett A., Seddon K.R., Wright R.D. (1979). Chemical modification of a titanium (IV) oxide electrode to give stable dye sensitisation without a supersensitiser. Nature.

