# Supplementary material for: Photoexcitation Processes in Oligomethine Cyanine Dyes for Dye-Sensitized Solar Cells—Synthesis and Computational Study
Source: Nanomaterials (Basel). 2020 Apr 2;10(4):662. doi: 10.3390/nano10040662 (PMC7221816; doi:10.3390/nano10040662)
Supplement: Supplementary file 1 [file nanomaterials-10-00662-s001.pdf]

nanomaterials

Supplementary Materials

Photoexcitation Processes in Oligomethine Cyanine Dyes for Dye-Sensitized Solar Cells—Synthesis and Computational Study

Corneliu I. Oprea [1], Petre Panait [2], Zahraa M. Essam [3], Reda M. Abd El-Aal [3,*] and Mihai A. Gîrțu [1,*]

[1] Department of Physics and Electronics, Ovidius University of Constanța, 900527 Constanța, Romania
[2] Doctoral School, Faculty of Physics, University of Bucharest, 077125 Bucharest, Romania
[3] Department of Chemistry, Suez University, 43511 Suez, Egypt
* Correspondence: mihai.girtu@univ-ovidius.ro (M.A.G); reda.abdelaal@suezuniv.edu.eg (R.M.A)

Contents of Supplementary materials:

1. **Synthesis of new oligomethine cyanine dyes**
    - Scheme S1, Scheme S2, Scheme S3—synthesis schemes
    - Figure S1–S22—FTIR and NMR spectra of compounds synthesized
    - Table S1—Characterization data for intermediate compounds and oligomethine cyanine dyes OMCD1–3
2. **Experimental absorption spectra of the oligomethine cyanine dyes**
    - Figure S23—Visible spectra of dyes OMCD1, OMCD2, and OMCD3 in ethanol
3. **Calculated electronic structure and absorption spectra of isolated oligomethine cyanine dyes**
    - Figure S24—Simulated UV-Vis spectra of isolated dyes
    - Figure S25—Matching with the solar spectrum of the absorption spectra of isolated dyes
    - Table S2—Wavelength, oscillator strength and composition of electronic transitions
4. **Calculated electronic structure and absorption spectra of adsorbed oligomethine cyanine dyes**
    - Figure S26—Electron density of adsorbed dyes
    - Table S3—Contributions of the building blocks to the electron density of the main molecular orbitals of the adsorbed dyes
    - Figure S27—Simulated UV-Vis spectra of adsorbed dyes
    - Figure S28—Matching with the solar spectrum of the absorption spectra of adsorbed dyes
    - Table S4—Wavelength, oscillator strength and composition of electronic transitions

1. Synthesis of new oligomethine cyanine dyes

Synthetic Concept: Commonly, asymmetrical oligomethine cyanine dyes **(OMCD 1–3)** have been synthesized via preparation of half dye intermediate **S1** 6-bromo-2-diethylidene 1,3,3-trimethyl-3H-benzo[e]indolinium-2yl 1,3,3-trimethyl indolinium-2yl-5-carboxy-iodide. The half dye **S1** is key starting material for synthesis of all **OMCD 1–3**. Reaction of equimolar ratio of **S1** with (4-diphenylphospho)phenyl)ethynyl **7** in the presence of catalyst, for example $Pd(OAc)_2$ /PPh_3,CuI/ Et_3N [15].,by adapting this reaction condition and stepwise reaction dehydrohalogenation via a half dye intermediate and compound **7**, unsymmetrical **OMCD 1** dye is achievable, **Scheme 1**.Reaction of equimolar amount of half dye intermediate **S1** and 7-(4-diphenylphosho)phenyl)ethynyl-3-yne-phenothiazine **S2** according to literature [16] afforded the corresponding **OMCD 2**, **Scheme 2**. Synthesis of **OMCD 3** was synthesized using Suzuki and Stille coupling routes. Starting with synthesis of 4,7-bis(5-bromo-5^{-}-ethynylthiophene-2,2^{-}-diyl)-benzo[c][1,2,5]thia- diazol **15**.,.which reacted with (4-diphenylphospho) phenyl) ethynyl **7** to give 4,7-bis(5^{-}-ethynyl)(4-

diphenylphospho)phenyl)ethynyl-thiophene-2,2'-diyl)-benzo[c][1,2,5]thiadiazole **16 (S3).** Reaction of equimolar amount of half dye intermediate **S1** and **S3** with stepwise dehydrohalogenation unsymmetrical **OMCD 3** dye is achievable, **Scheme 3**.

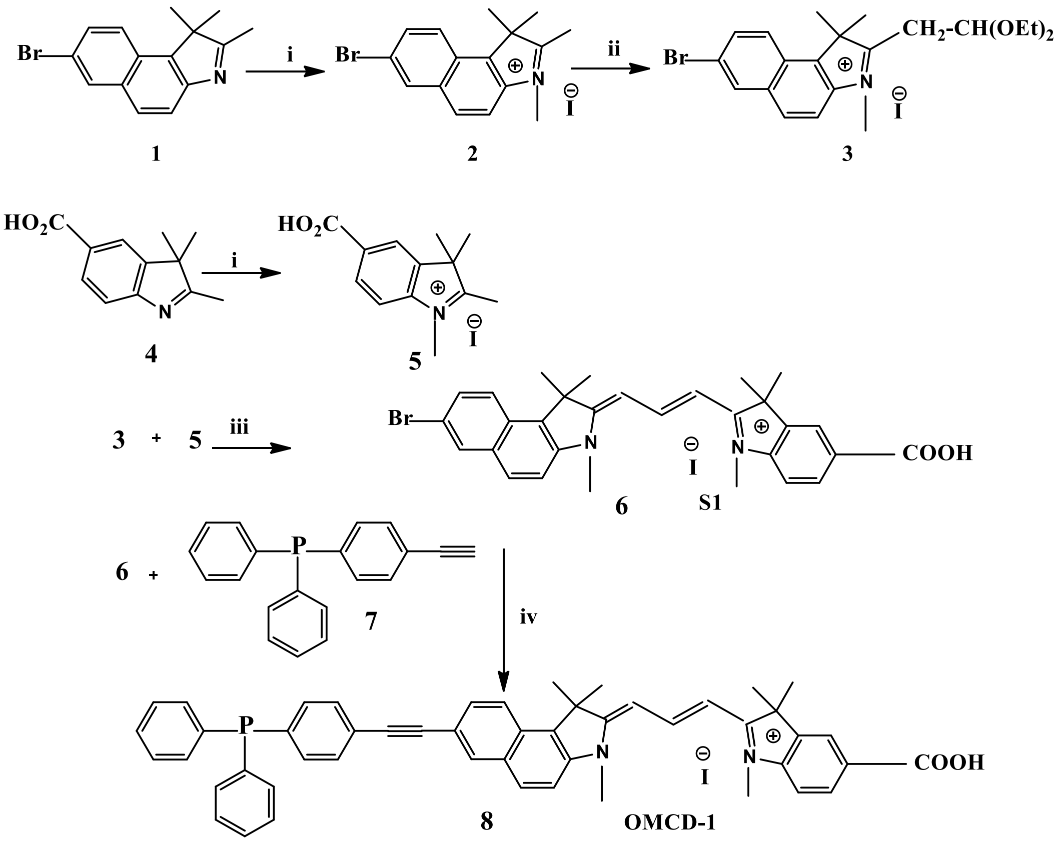

i- MeI ii- triethoxymethan, EtOH/pip. iii- EtOH/pip iv- $Pd(ACO)_2$,PPh_3,Et_3N/CuI

Scheme 1

Synthesis of 6-bromo-1,2,3,3-tetramethyl-3H-benzo[e]indolinium-2yl iodide (2)

Compound 1 (7g) reacted with excess amount of methyliodide.The reaction mixture was refluxed gently for 2 hours. The precipitated product was collected, washed with ether and dried to give compound 2, 5.3 g of red solid was obtained. ^{1}H NMR (DMSO,400 MHz, TMS) δ 7.79 (m, 5H), 3.43 (s, 3H), 2.63 (s, 3H), 1.45 (s, 6H). Mass: m/z calcd for $C_{16}H_{15}NBrI$ ($[M+2H]^+$) 428, found 430.

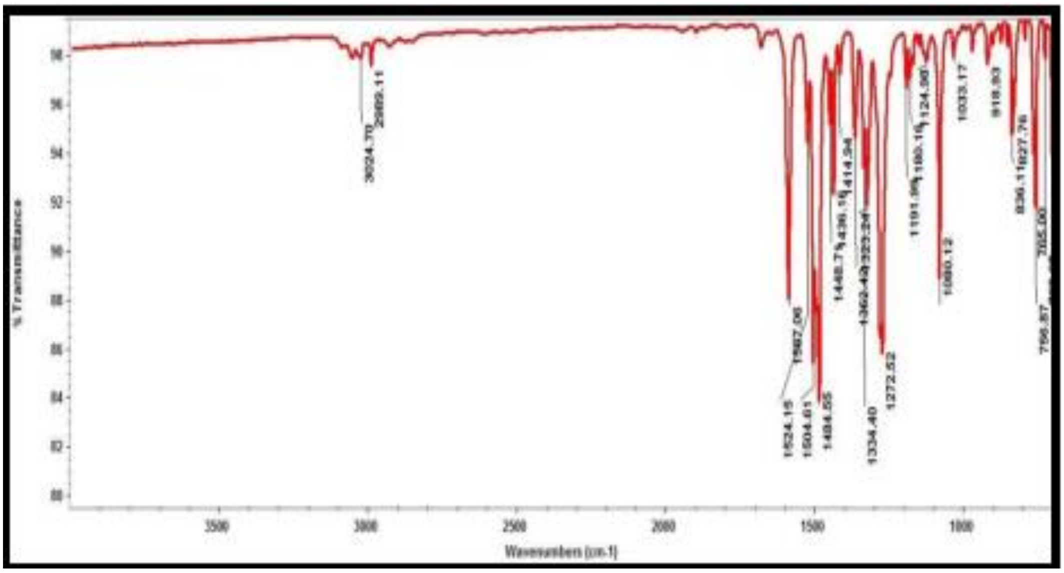

Figure S1. FT-IR spectra for compound 2.

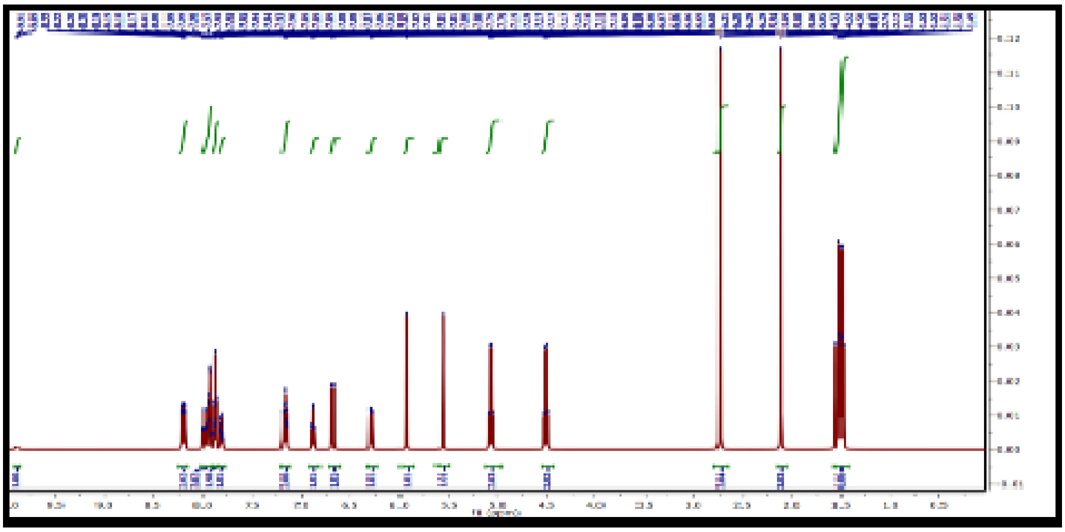

Figure S2. ^{1}H-NMR spectrum for compound 2.

Synthesis of 6-bromo-2-(2,2-diethoxyethyl)-1,2,3,3-tetramethyl-1H-benzo[e]indolinium-2yl iodide (3).

Compound **3** was prepared by reaction of compound **2** (0.428 g, 0.00mmol) with ethylortho formate (0.184 g, 0.004mmol) in ethanol as solvent, purchased by piperidine as basic catalyst. The reaction mixture was refluxed for 7 hours, filtered hot, concentrated, cooled and neutralized by acetic acid. The solid product was collected and recrystallized from ethanol to give compound **3**,asbrown solid 79.5%.M.p=79-81°C.. ^{1}H NMR (DMSO,400 MHz) δ 7.79 (m, 5H), 3.43 (s, 3H), 2.63 (s, 3H), 1.45 (s, 6H). Mass: m/z calcd for $C_{21}H_{23}NO_2BrI$ ([M + H]$^+$) 528, found 529.

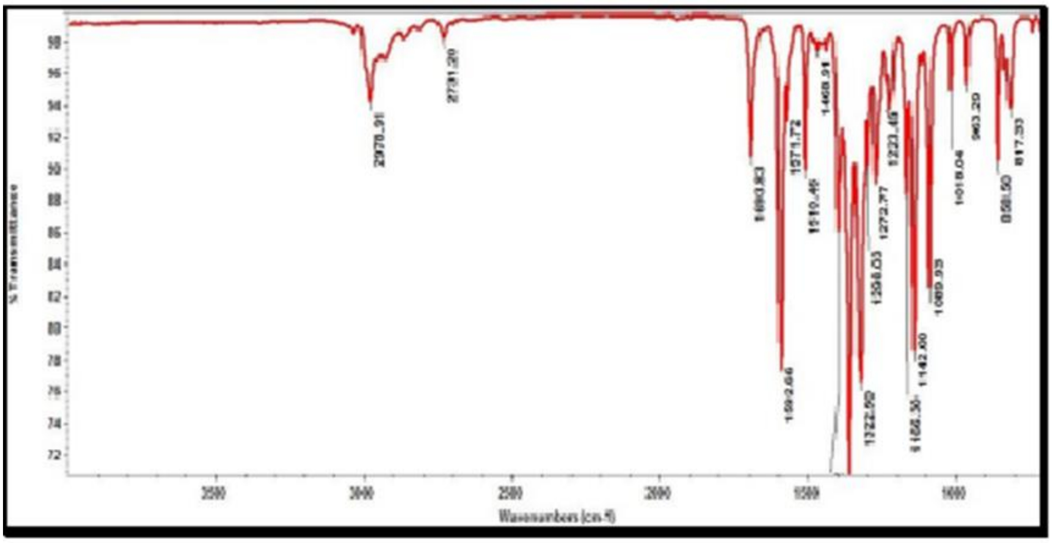

Figure S3. FT-IR spectra for compound 3.

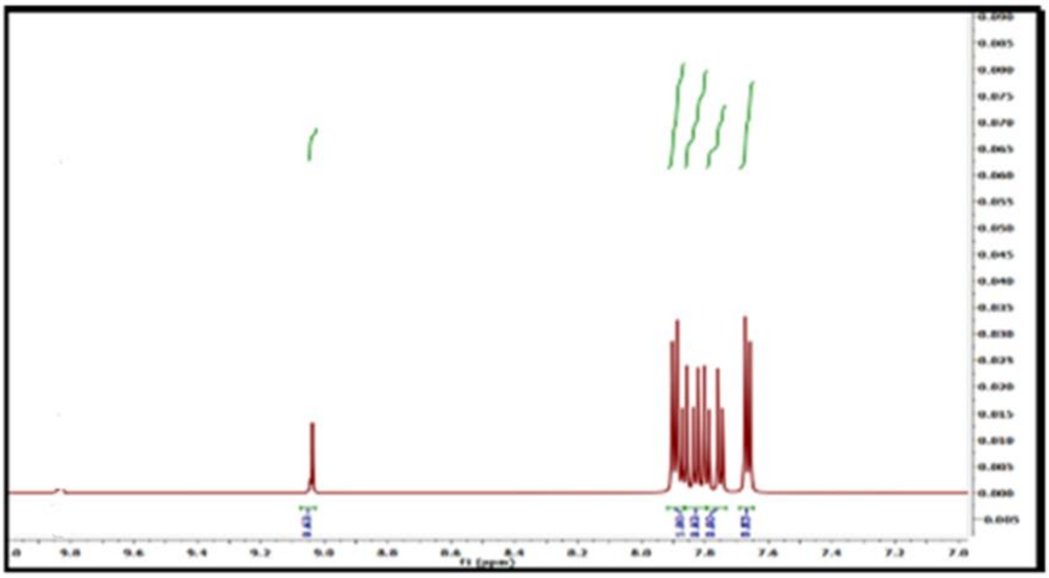

Figure S4. ^{1}H-NMR spectrum for compound 3.

Synthesis of -1,2,3,3-tetramethyl-3H-indolinium-2yl-5-carboxy iodide (5)

Compound **5** was prepared in the same manner for compound **2**. yield:77%. Mp: 71–73 °C. ^{1}H NMR (DMSO,400 MHz) δ 12.98 (s, 1H),7.67-7.50 (m, 3H), 3.47 (s, 3H), 2.15 (s, 3H), 1.55 (s, 6H). APCI-mass: m/z calcd for $C_{13}H_{16}NO_2I$ ([M + H]$^+$) 345, found 346.

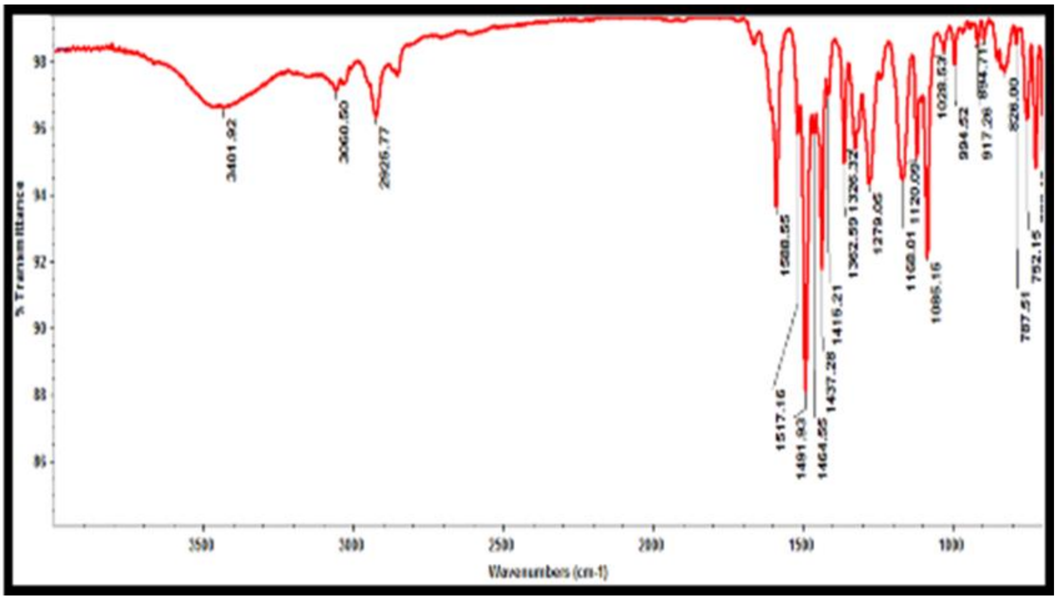

Figure S5. FT-IR spectra for compound 5.

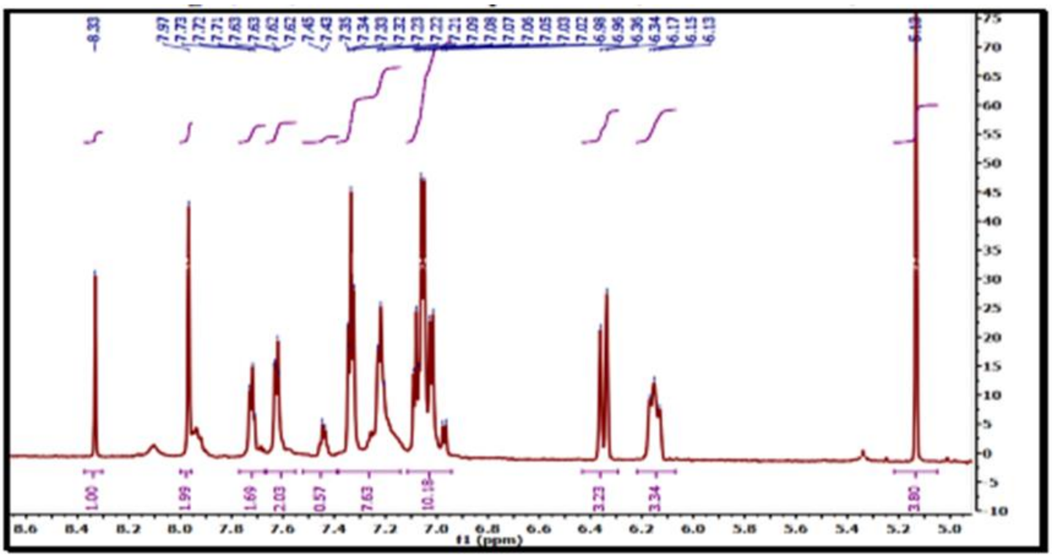

Figure S6. ^{1}H-NMR spectrum for compound 5.

Synthesis of 6-bromo-2-diethylidene 1,3,3-trimethyl-3H-benzo[e]indolinium-2yl 1,3,3-trimethyl indolinium-2yl-5-carboxy-iodide (6).

A mixture of equimolar amounts of compound **3** (0.002mol) and compound **5** (0.002mol) dissolved in ethanol and catalyzed by piperidine (0.5 mL). The reaction mixture was refluxed for 11 hours. The product was filtered hot, concentrated, cooled and neutralized by acetic acid (0.3mL). The precipitated compound was recrystallized from ethanol to give compound **6**, yield:87.75%. Mp: 151–153 °C. ^{1}H NMR (DMSO,400 MHz) δ 12.98 (s, 1H),7.69-7.55 (m, 8H), 6.78 (d, 1 H, J =7.4 Hz), 6.67 (t, 1 H, J =7.3 Hz), 6.50 (d, 1 H, J =8.4 Hz), 3.86 (s, 3H), 2.10 (s, 3 H), 1.50–1.40 (m, 12 H). ([M + 2 H]$^{+}$) 657, found 659.

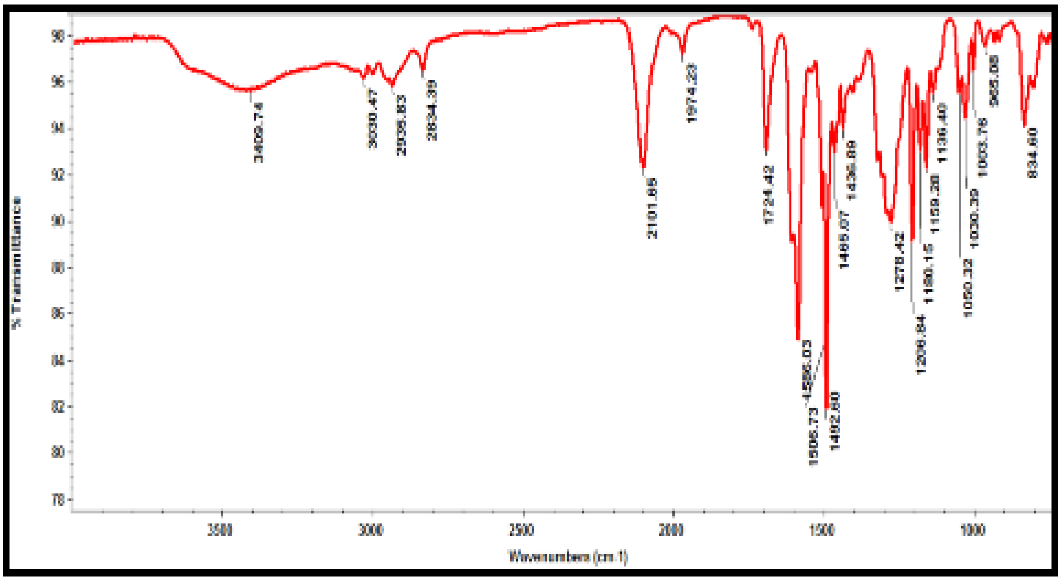

Figure S7. FT-IR spectra for compound 6.

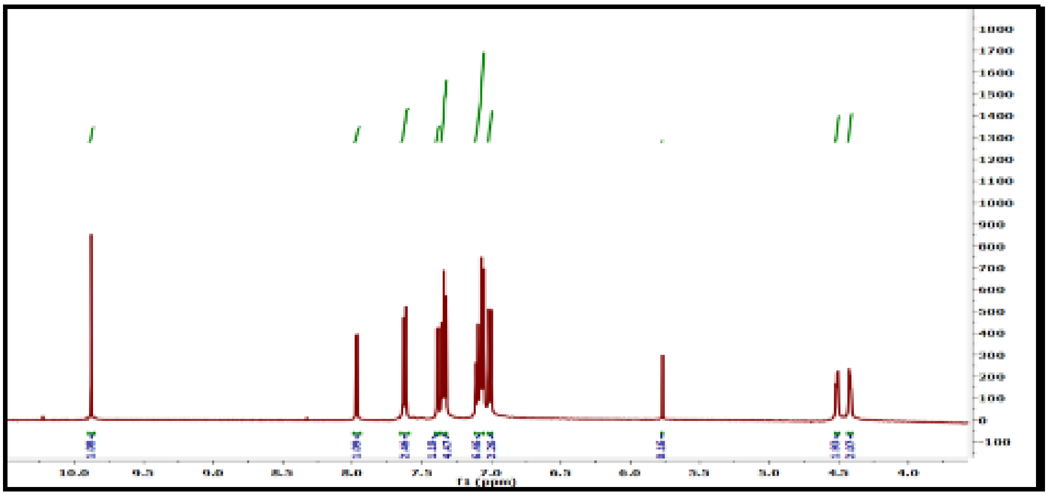

Figure S8. ^{1}H-NMR spectrum for compound 6.

Synthesis of 2-(7-(4-diphenylphospho)phenyl)ethynyl-1,1,3-trimethyl-1H-benzo[e]indol-2(3H)-yliedene)prop-1-ene-1-yl)-5-carboxy-1,3,3,-trimethyl-3H-indolin-1-ium iodide-OMCD1.(8).

To a degassed solution of **6** (0.577 g mmol) in dry Et_3N (10 mL) and THF (3 mL) were successively added $Pd(OAc)_2$ (15.5 mg, 0.07 mmol), PPh_3 (14.5 mg, 0.06 mmol),CuI (10.5 mg, 0.06 mmol) and (4-diphenylphospho)phenyl)ethynyl **7** (0.592. g, 2.07 mmol). The reaction mixture was refluxed under nitrogen for 6 h. After removal of the solvent, the residue was purified by column chromatography (silica gel, DCM/petroleum ether, 3/1, V/V). 270 mg of yellow solid was obtained, yield:87.0%. Mp: 131–132 °C. ^{1}H NMR (DMSO,400 MHz) δ 12.79 (s, 1 H-COOH), 7.82 (d, 2 H, J=8.4 Hz), 7.63 (d, 2 H, J=8.4 Hz), 7.56 (s, 1 H), 7.50 (d, 2 H, J=7.4 Hz), 7.49–7.51 (m, 15 H), 6.88 (d, 1 H, J =7.4 Hz), 6.80 (t, 1 H, J =7.3 Hz), 6.50 (d, 1 H, J =8.4 Hz), 3.86 (s, 3H), 2.15 (s, 3 H), 1.52–1.41 (m, 12 H). Mass: m/z calcd for $C_{50}H_{44}N_2O_2PI$ ([M + H]$^+$) 862, found 863.

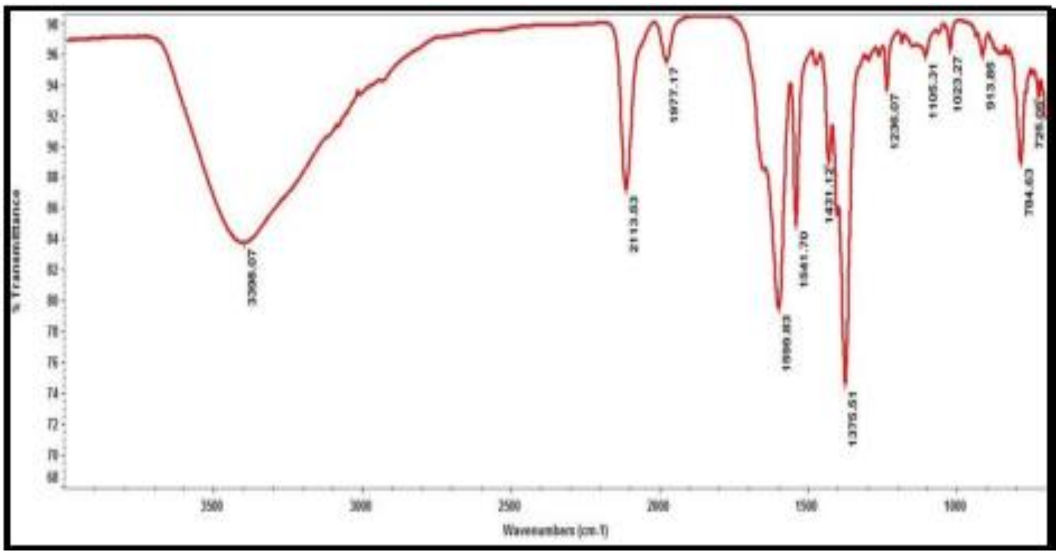

Figure S9. FT-IR spectra for compound 8.

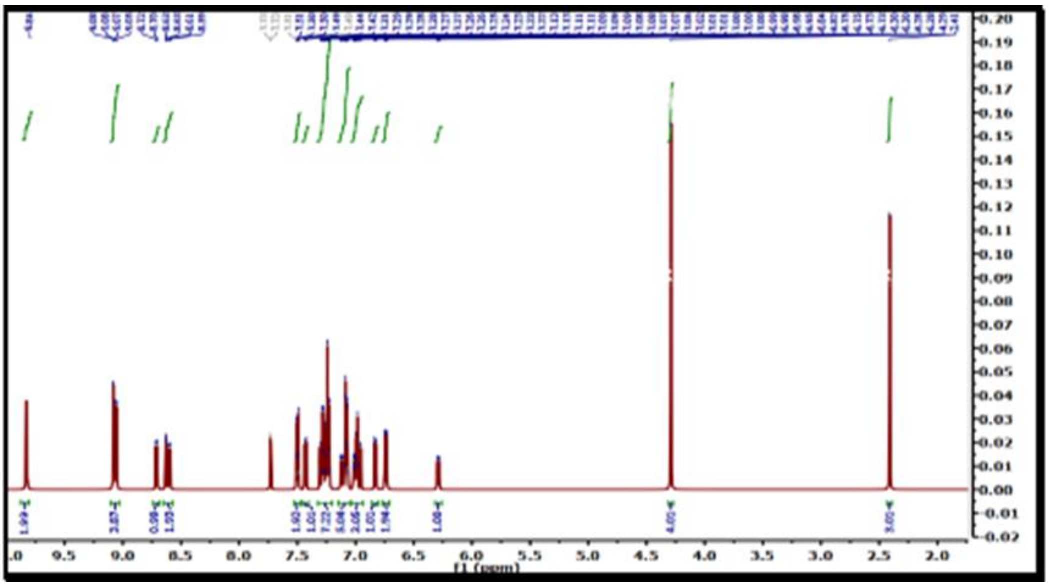

Figure S10. ^{1}H-NMR spectrum for compound 8.

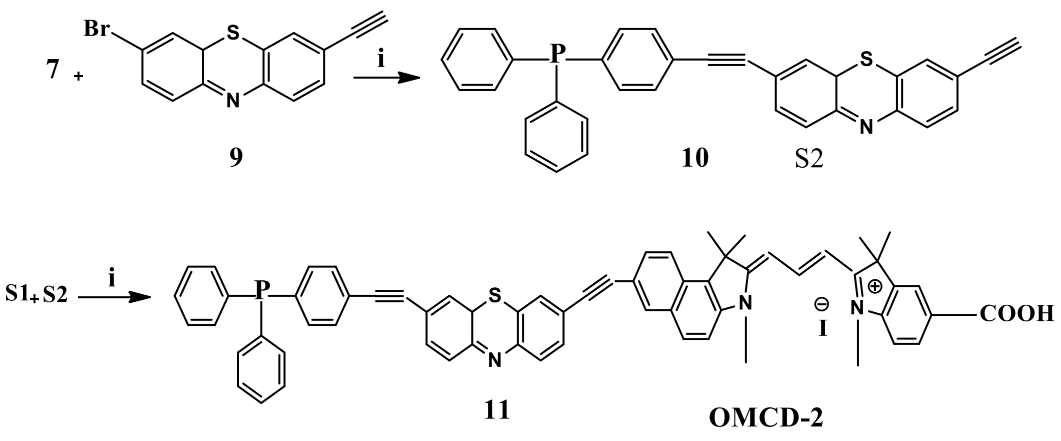

i- $Pd(PPh_3)_4$,CuI, diisoPro.amine, THF

Scheme 2

Synthesis 7-(4-diphenylphosho)phenyl)ethynyl-3-yne-phenothiazine 10.

Pd(PPh3)4 (0.014 g, 0.012 mmol), CuI (0.008 g, 0.04 mmol) were added into 15 mL di-isopropylamine under an argon atmosphere and cooled to 0°C. Then compound **7** (0.215 g, 0.6 mmol) and 7-bromo-3-yne-phenothiazine **9** (0.003 g mmol) in tetrahydro- furan (0.84 mL, 0.84 mmol) were added to the mixture. The reaction mixture was stirred at room temperature for 6 hours, and then the solution was poured into 150 mL water and extracted by DCM. Solvents were removed in vacuum and the residue was purified by column chromatography on silica gel (hexane:DCM = 5:1) affording white solid of target product **10**, yield 62%. M.p= 190-192 °C.

^{1}H NMR (DMSO, 400 MHz) δ ppm 7.73-7.61 (m, 6H-Ar-H), 7.59-7.11 (m, 14H-Ar-H), 6.57 (s, 1H). APCI-mass: m/z calcd for $C_{34}H_{30}NSP$ ([M + H]$^+$) 515, found 515.

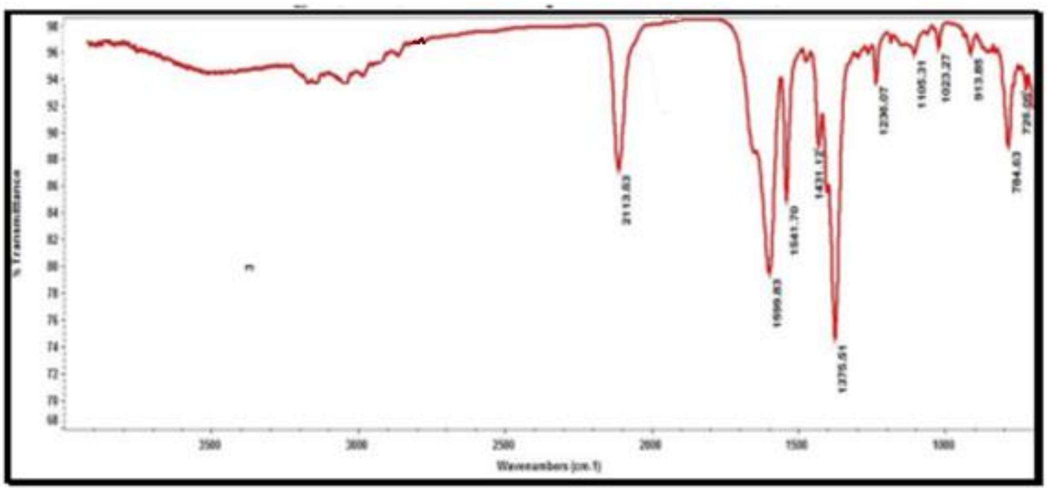

Figure S11. FT-IR spectrum for compound 10.

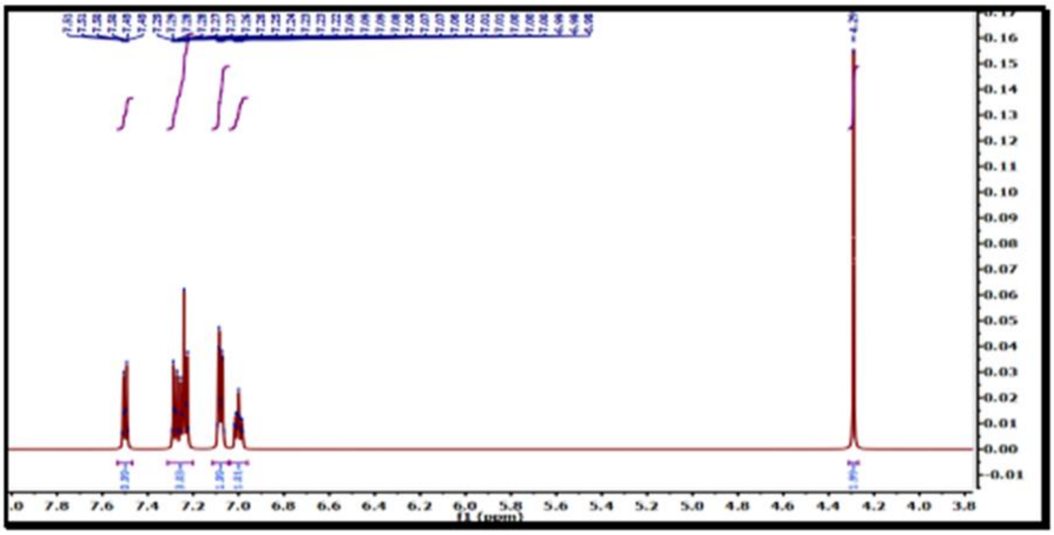

Figure S12. ^{1}H-NMR spectrum for compound 10.

Synthesis of 7-(4-diphenylphosho)phenyl)ethynyl-3-yne-(7(1,1,3-trimethyl-1H-benzo[e]indolinium-2(3H)-yliedene)prop-1-ene-1-yl)-5-carboxy-1,3,3,-trimethyl-3H-indolin-1-ium iodide-phenothiazine 11 (OMCD 2).

Pd(PPh3)4 (0.014 g, 0.012 mmol), CuI (0.008 g, 0.04 mmol) were added into 15 mLdi-isopropylamine under an argon atmosphere and cooled to 0°C. Then compound **10** (0.304 g, 0.6 mmol) and compound **6** (0.577 g mmol) in tetrahydrofuran (0.84 mL, 0.84 mmol) were added to the mixture. The reaction mixture was stirred at room temperature for 6 hours, and then the solution was poured into 150 mL water and extracted by DCM. Solvents were removed in vacuum and the residue was purified by column chromatography on silica gel (hexane:DCM = 5:1) affording white solid of target product **11**, yield 62%, M.p=155-157 °C. ^{1}H NMR (DMSO, 400 MHz) δ ppm 12.97 (s, 1H),7.83-7.65 (m, 27H-Ar-H), 6.88 (d, 1 H, J =7.4 Hz), 6.80 (t, 1 H, J =7.3 Hz), 6.50 (d, 1 H, J =8.4 Hz), 3.86 (s, 3H), 2.15 (s, 3 H), 1.52–1.41 (m, 12 H). Mass: m/z calcd for $C_{64}H_{59}N_3O_2SPI$ ([M + 3H]$^+$) 1091, found 1093.

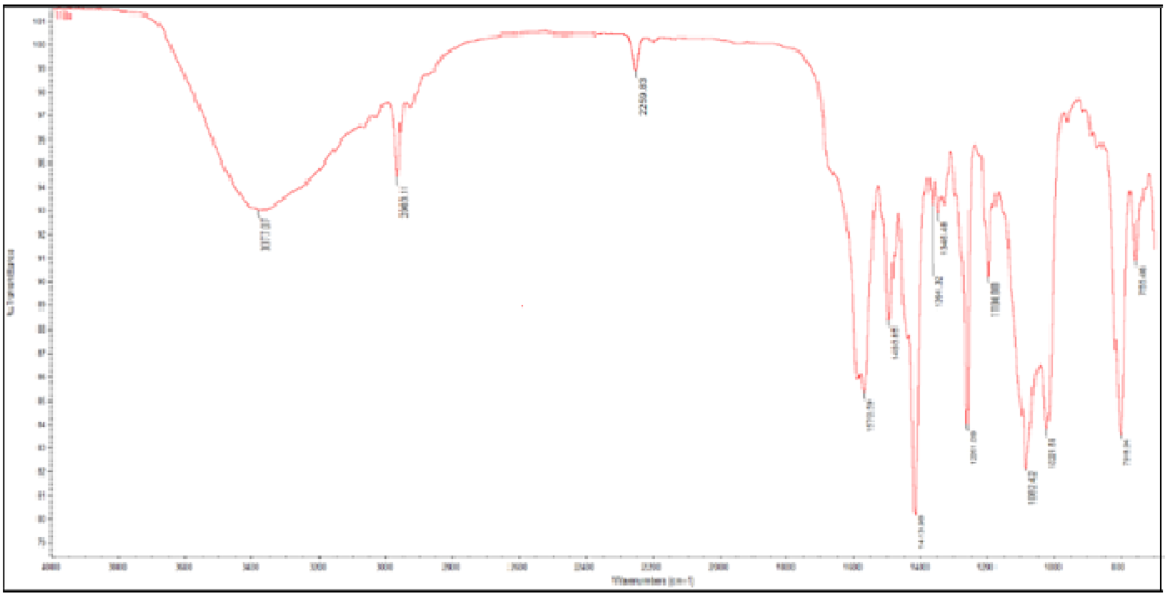

Figure S13. FT-IR spectrum for compound 11.

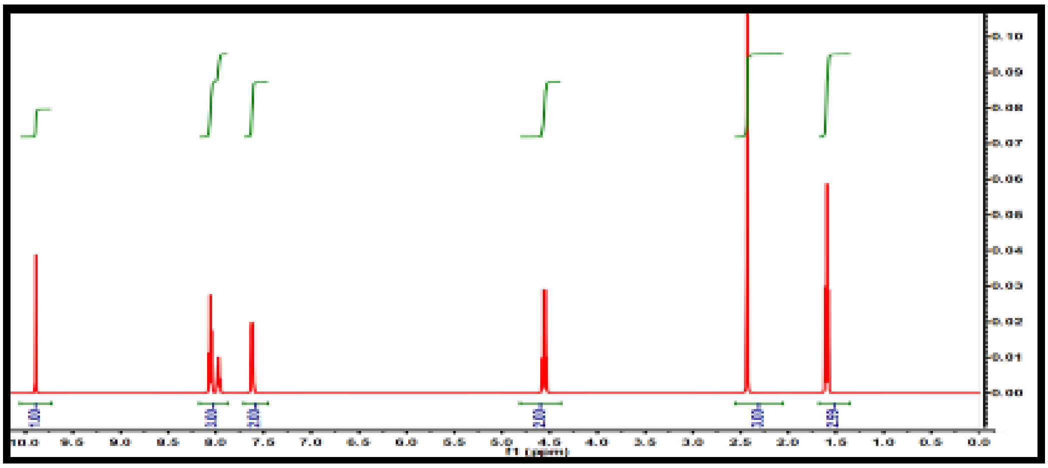

Figure S14. ^{1}H-NMR spectrum for compound 11.

Synthesis of 5-bromo-(2-tributyl-thiophene-2yl)stannane 13.

A solution of N-bromosuccinimide (NBS) (1.48 g, 8.31 mmol) in DMF (10 ml) was added dropwise to a solution of 2-Tributyl-(thiophene-2-yl)stannane **12** (2.48 g, 6.65 mmol) with exclusion of ambient light and the reaction mixture was stirred for 72 h at 60 C. The mixture was poured into water and extracted with dichloromethane. The organic extract was dried over magnesium sulfate and the solvent removed under reduced pressure. The crude product was purified by column chromatography eluting with hexane : dichloromethane to give the product **13** (1.75 g, 64%) as bright deep yellow solid.

^{1}H NMR (DMSO, 400 MHz) δ 7.62 (d, 1H, Th), 7.86 (d, 1H, Th), 3.89 (d, 6H, 3 CH2), 2.45-2.30 (m,6H 3CH$_2$), 1.74–1.50 (m, 6H, 3CH2), 1.15 (t, 9H, 3CH$_3$). Mass: m/z calcd for $C_{16}H_{29}SBrSnI$ ($[M + H]^+$) 541.71, found 542.

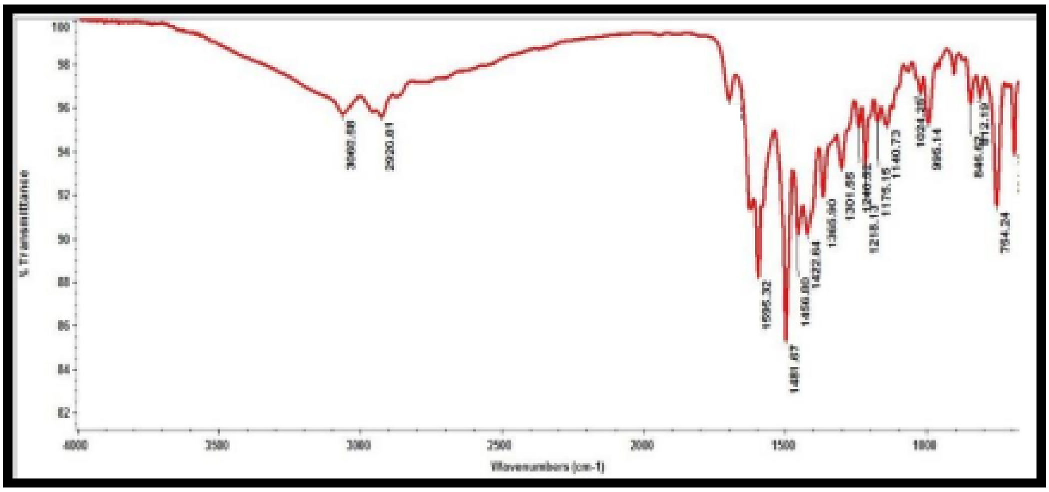

Figure S15. FT-IR spectrum for compound 13.

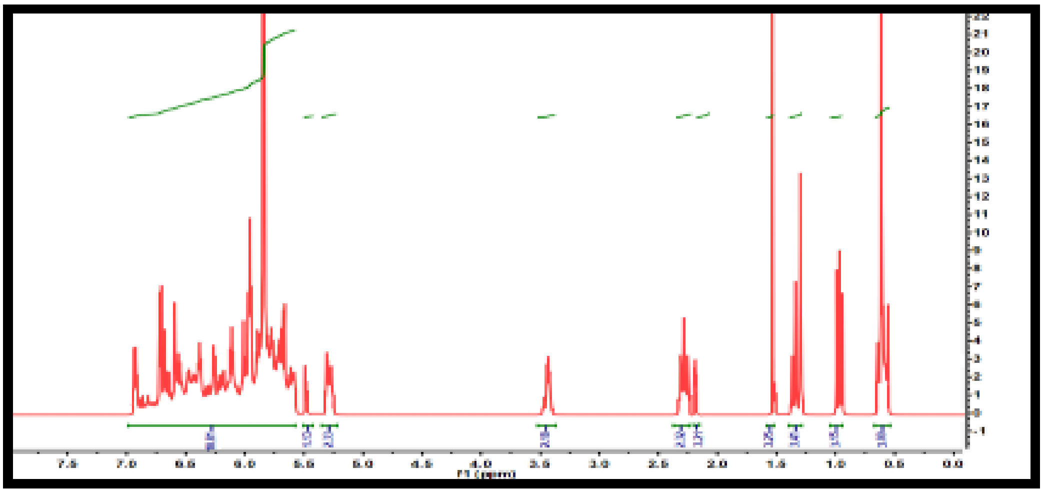

Figure S16. ^{1}H-NMR spectrum for compound 13.

Synthesis of 4,7-bis(5,5′-dibromothiophene-2,2′-diyl)-benzo[c][1,2,5]thiadiazol 14.

5-Bromo-2-tributyl-(thiophene-2-yl)stannane **13** (9.29 g, 20.56 mmol),4,7-dibromobenzo [c][1,2,5]thiadiazole (2.35 g, 8 mmol) and $Pd(PPh_3)4$ (0.2 mmol, 0.231 mg) were added to a round bottom Schlenk flask and kept under vacuum for 15 min. DMF (30 ml) was then added in to the above mixture under argon and stirred for another 15 min. The solution was subjected to three vacuum/argon refill cycles and then heated at 100 °C with vigorous stirring for 72 h under argon. Reaction completion was confirmed by MALDI-TOF and TLC. The mixture was then poured into water and extracted with dichloromethane. The organic layer was washed three times with water and dried over $MgSO_4$. The crude product was purified using column chromatography (silica gel, hexane : dichloromethane as eluent) followed by precipitation in dichloromethane / methanol to yield the product as an orange solid,yield 65%. M.p= 115-117 °C. ^{1}H NMR (DMSO, 400 MHz) δ 7.79 (d, 2H Ar-H), 6.85 (d, 4 H, Th) Mass: m/z calcd for $C_{14}H_6N_2S_3Br_2$ ($[M+1 H]^+$) 458, found 459.

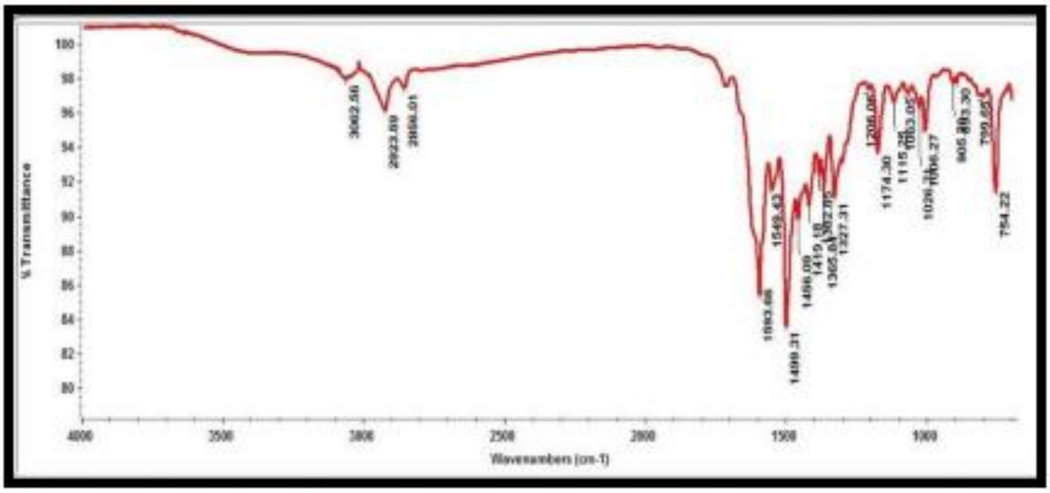

Figure S17. FT-IR spectrum for compound 14.

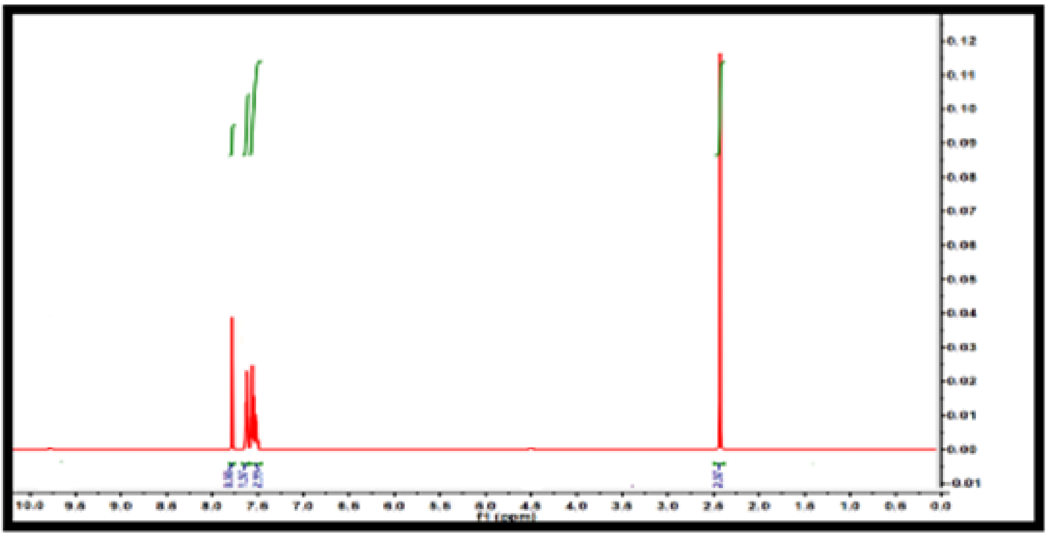

Figure S18. ^{1}H-NMR spectrum for compound 14.

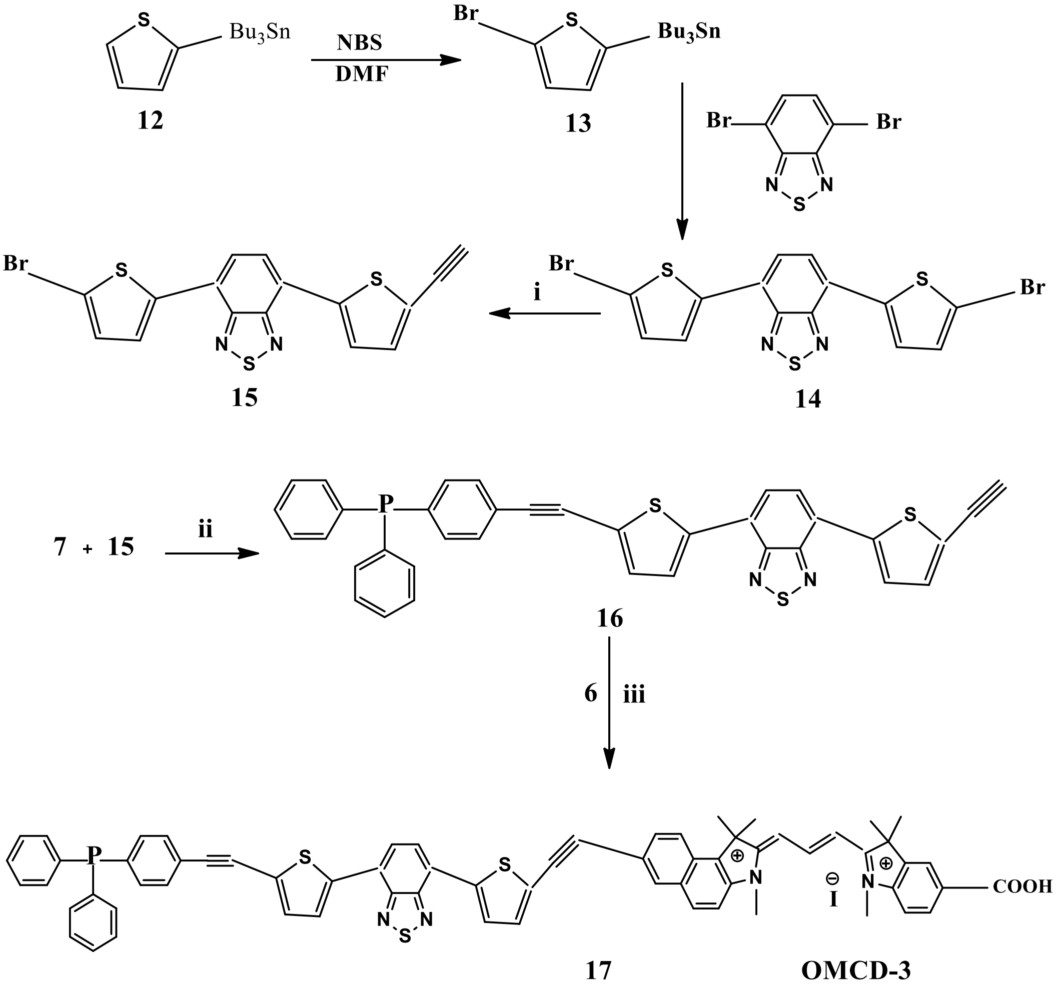

i- Pd(PPh_3)$_4$,DMF, ii- Pd((PPh_3)$_2$$Cl_2$, (Me)$_3$SiCCH, PPh_3, CuI,Et_3N, THF,
iii- Pd(PPh_3)$_4$,CuI, diisoPro.amine, THF

Schem-3

Synthesis of 4,7-bis(5-bromo-5'-ethynylthiophene-2,2'-diyl)-benzo[c][1,2,5] thiadiazol 15.

To a degassed solution of **14** (1.48 g, 4.43 mmol) in dry EtN_3 (8 mL) and THF (5 mL) were successively added $Pd(PPh_3)_2Cl_2$ (124.0 mg, 0.17 mmol), PPh_3 (46.5 mg, 0.17 m mol), and CuI (33.7 mg, 0.17 mmol) and ethynyltrimethylsilane (435.0 mg, 4.43 m mol).The reaction mixture was refluxed under nitrogen for 6 h. Then K_2CO_3 (1.65 g, 12 mmol) and methanol (5 mL) were added and the solution was stirred for 1 h at room temperature. The solvents were removed. The residue was taken up with DCM and washed with water. The organic layer was dried over $MgSO_4$. After the solvent was removed, the residue was purified by column chromatography (silica gel, DCM/ petroleum ether, 1/6, V/V). 1.04 g of yellow liquid was obtained, yield: 84.0%.

^1H NMR (DMSO, 400 MHz) δ 7.75 (d, 2H Ar-H), 7.45 (s, 1H), 6.85 (d, 4 H, Th). Mass: m/z calcd for $C_{16}H_7N_2S_3Br$ ($[M+2H]^+$) 403, found 405.

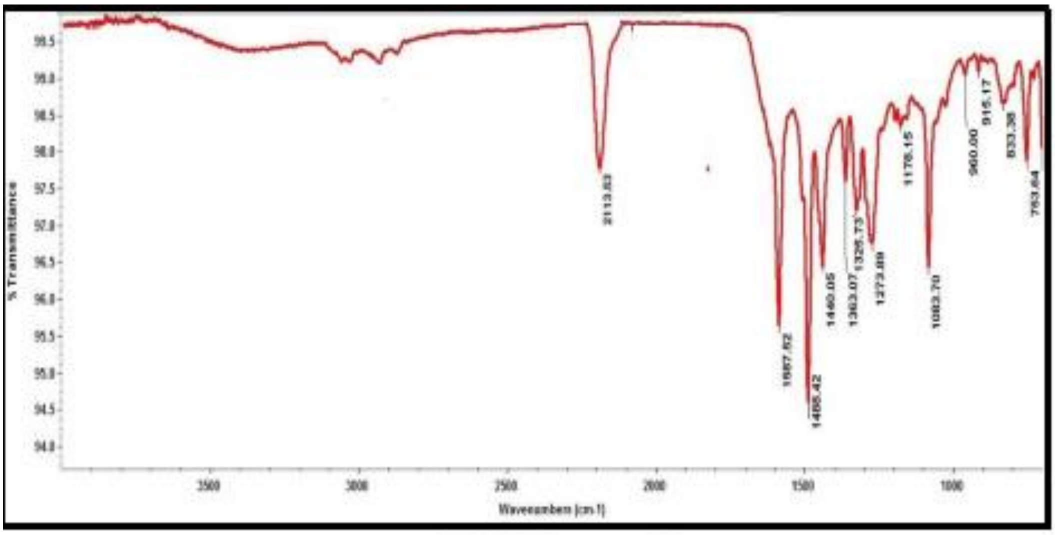

Figure S19. FT-IR spectrum for compound 15.

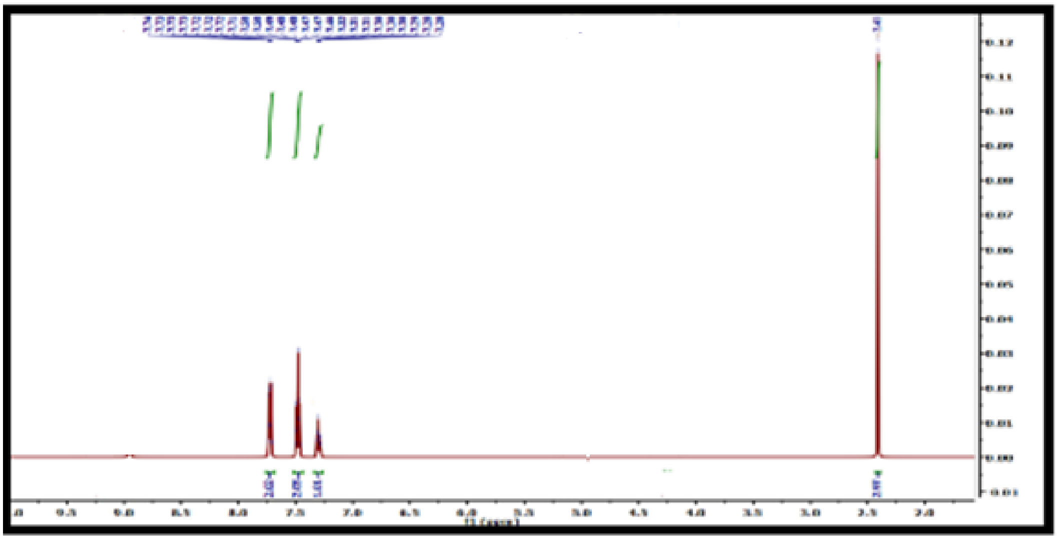

Figure S20. ^1H-NMR spectrum for compound 15.

Synthesis of 4,7-bis(5′-ethynyl)(4-diphenylphospho)phenyl)ethynyl-thiophene-2,2′-diyl)-benzo[c] [1,2,5] thiadiazol 16.

Compound **16** was prepared in same manner for compound **10,** yield 77%. M.p=145-147 °C. ^{1}H NMR (DMSO, 400 MHz) δ 7.75-7.65 (m, 16H-Ar-H), 7.60-7.45 (m, 4H-Ar-H), 6.80 (s, 1H) Mass: m/z calcd for $C_{36}H_{21}N_2S_3P$ ([M + H]$^+$) 608, found 608.

Synthesis of 4,7-bis(5′-(4-diphenylphospho)phenyl)ethynyl-5-yne-thiophene-2,2′-diyl)(1,1,3-trimethyl-1H-benzo[e]indolinium-2(3H)-yliedene)prop-1-ene-1-yl)-5-carboxy-1,3,3,-trimethyl-3H-indolin-1-ium iodide-phenothiazine-benzo[c][2,1,3]thiadiazol (OMCD 3).17.

Pd(PPh$_3$)4 (0.014 g, 0.012 mmol), CuI (0.008 g, 0.04 mmol) were added into 15 mLdi-isopropylamine under an argon atmosphere and cooled to 0 °C. Then compound **16** (0.365 g, 0.6 mmol) and compound **6** (0.577 g 1 mmol) in tetrahydrofuran (0.84 mL, 0.84 mmol) were added to the mixture. The reaction mixture was stirred at room temperature for 6 hours, and then the solution was poured into 150 mL water and extracted by DCM. Solvents were removed in vacuum and the residue was purified by column chromatography on silica gel (hexane:DCM = 5:1) affording white solid of target product **17**, yield 67%. M.p=175-177 °C. ^{1}H NMR (DMSO,400 MHz) δ ppm 12.98 (s. 1H), 7.95-7.87 (m, 24H-Ar-H, Btz), 7.61-7.50 (m, 4H-Ar-H, Th), 6.88 (d, 1H, J =7.3 Hz), 6.83 (t, 1H, J =7.4 Hz), 6.50 (d, 1H, J =7.3 Hz), 3.87 (s,3H), 2.15 (s, 3 H), 1.53–1.40 (m, 12H). Mass: m/z calcd for $C_{66}H_{50}N_4S_3O_2PI$ ([M +1 H]$^+$) 1184, found 1185.

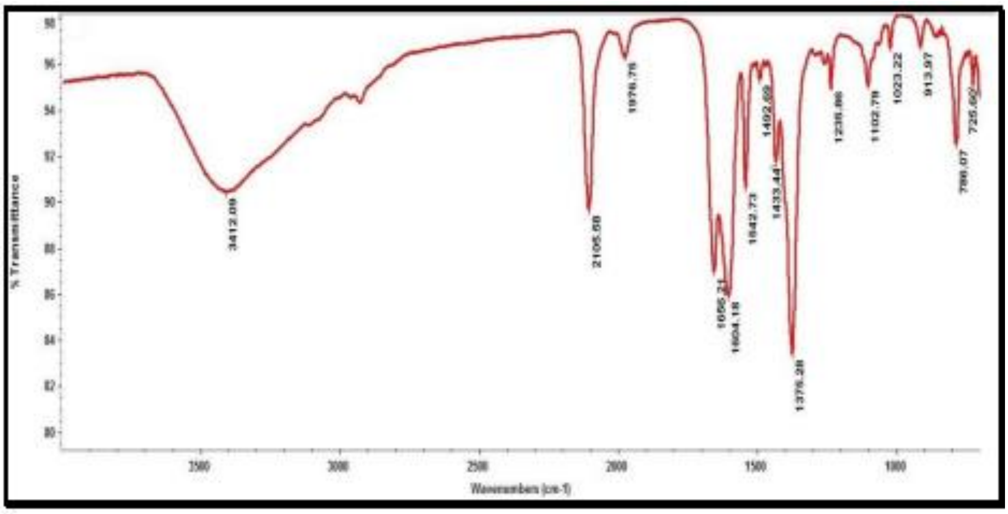

Figure S21. FT-IR spectrum for compound 17.

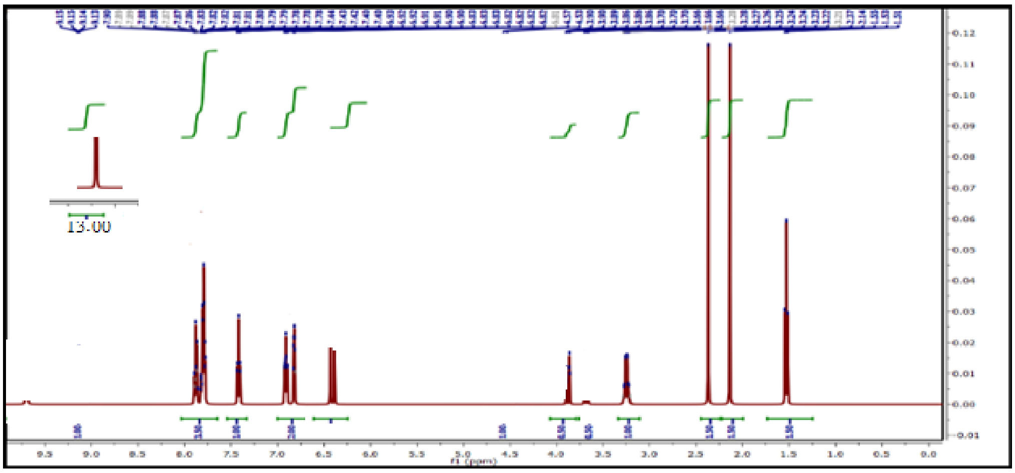

Figure S22. ^{1}H-NMR spectrum for compound 17.

Table S1. Characterization data for compounds (2-17) and oligomethine cyanine dyes OMCD 1–3.

Compd. No	Nature of products				Analysis % Calcd.		(Found)	
	M.p. (°C)	Yield (%)	Color	Mol.Formula (Mol.wt.)	C	H	N	S
2	53	75.71	Yellow	$C_{16}H_{17}NBrI$ (430)	44.65 (44.03)	3.95 (3.89)	3.26 (2.97)	-
3	81	79.50	Orange	$C_{21}H_{27}NO_2BrI$ (532)	47.37 (47.13)	5.08 (4.81)	2.63 (2.81)	-
5	73	77	Yellow	$C_{13}H_{16}NO_2I$ (345)	45.22 (44.97)	4.64 (4.33)	4.06 (3.87)	-
6	153	87	Red	$C_{30}H_{30}N_2O_2BrI$ (657)	54.80 (55.09)	4.57 (4.35)	4.26 (4.39)	-
8	131	79	Deep violet	$C_{50}H_{44}N_2O_2PI$ (862)	69.61 (69.99)	5.10 (4.89)	3.25 (3.43)	
10	191	65	Orange	$C_{34}H_{21}NSP$ (506)	80.63 (80.47)	4.15 (4.45)	2.77 (3.07)	6.21 (5.89)
11	155	63	Intense violet	$C_{64}H_{50}N_3O_2SPI$ (1082)	70.80 (71.09)	4.62 (4.33)	3.88 (4.11)	2.96 (3.15)
13	133	67	Red	$C_{16}H_{29}SBrSn$ (414.7)	46.30 (46.67)	6.99 (7.17)	----	7.72 (7.55)
14	117	65	Yellow	$C_{14}H_6N_2S_3Br_2$ (458)	36.68 (36.99)	1.31 (1.55)	6.11 (5.89)	20.96 (19.78)
15	137	85	Reda	$C_{16}H_7N_2S_3Br$ (403)	47.64 (47.33)	1.74 (2.09)	6.95 (7.15)	23.82 (23.57)
16	145	87	Deep red	$C_{36}H_{21}N_2S_3P$ (608)	71.05 (70.87)	3.45 (3.77)	4.61 (4.37)	15.79 (15.99)
17	177	67	Deep violet	$C_{66}H_{50}N_4S_3O_2PI$ (1184)	66.89 (67.11)	4.22 (3.97)	4.73 (4.55)	8.11 (8.47)

2. Experimental absorption spectra of the oligomethine cyanine dyes

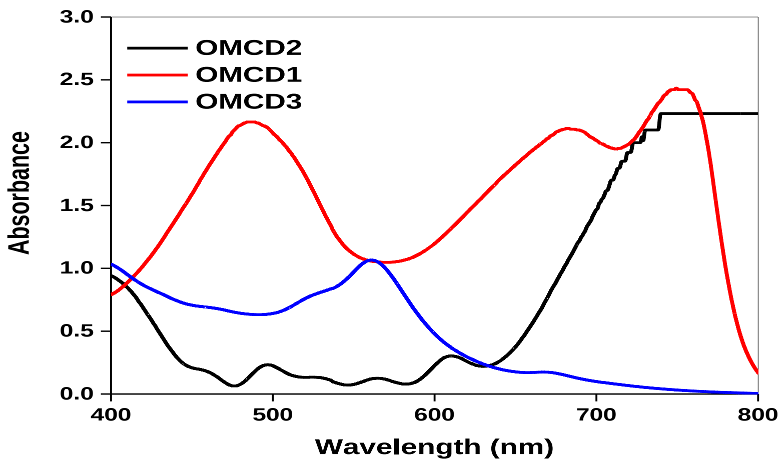

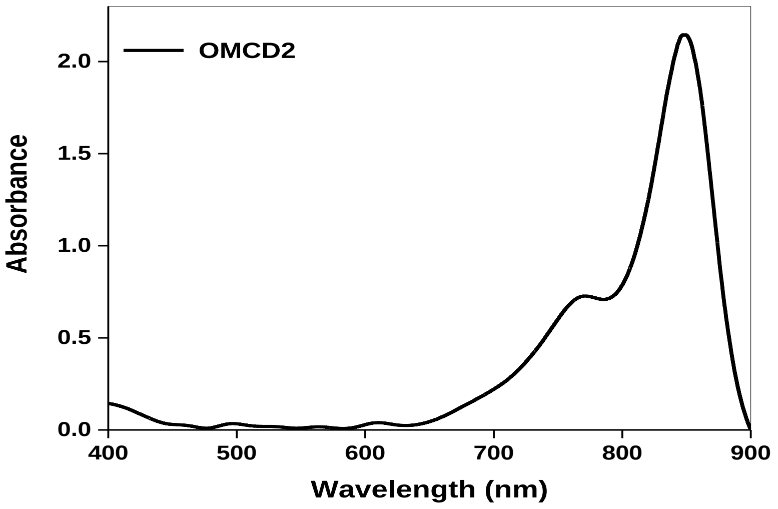

Figure S23. Visible spectra of dyes OMCD, OMCD2, and OMCD3 in ethanol (1.0 × 10^{-4} M) (top) and of OMCD2 (1.0 × 10^{-5} M) (bottom).

3. Calculated electronic structure and absorption spectra of the oligomethine cyanine dyes

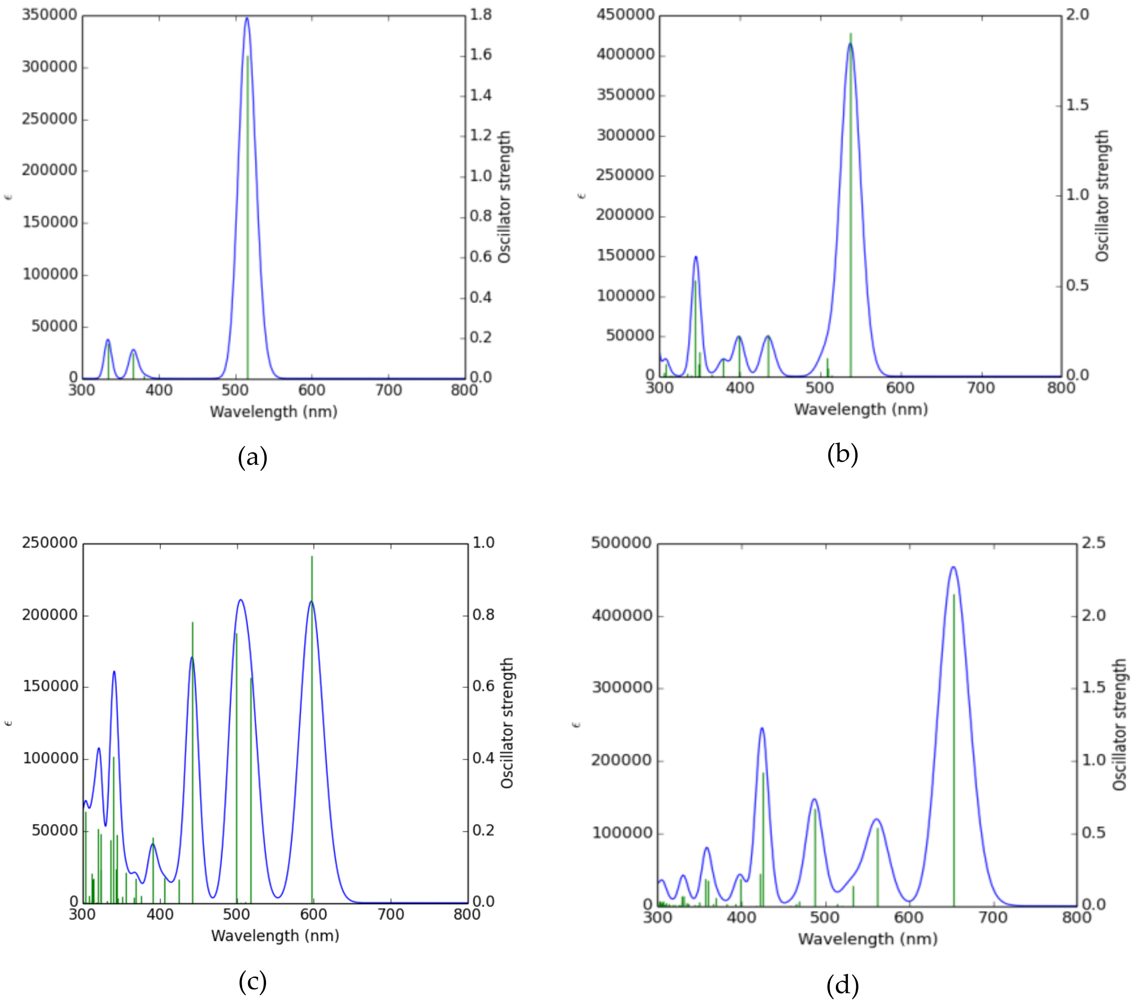

Figure S24. Simulated UV-Vis spectra by DFT calculation for the isolated dyes NKX-2311 (**a**), OMCD1 (**b**), OMCD2 (**c**) and OMCD3 (**d**) in ethanol. The spectral lines were convoluted with Gaussian distributions of 1000 cm^{-1} linewidth at half maximum.

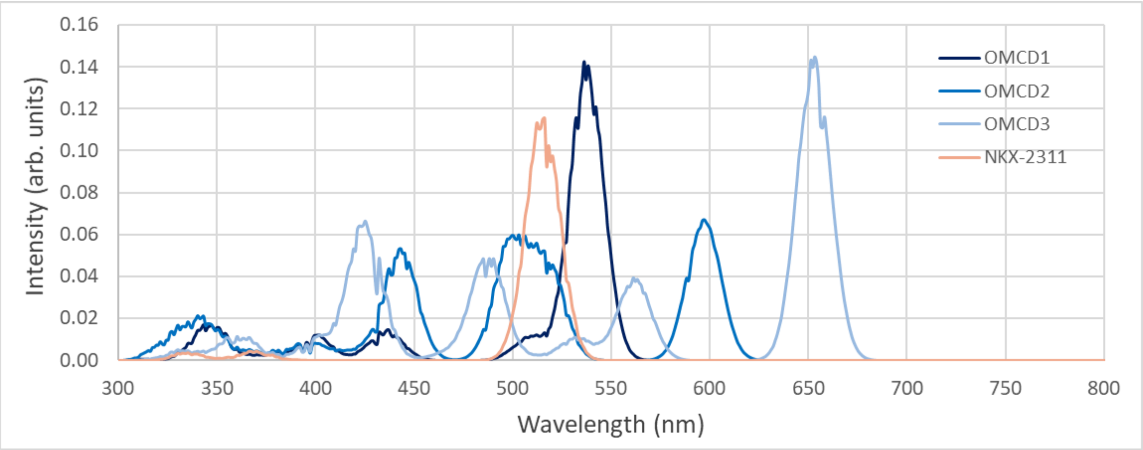

Figure S25. Simulated UV-Vis spectra by DFT calculation for the isolated dyes weighted with the standard solar irradiance.

Table S2. Wavelength, oscillator strength, and composition of the main electronic transitions for OMCD1, OCD2 and OMCD3.

Dye	Wavelength (nm)	Oscillator Strength	Major contributions
OMCD1	538	1.9037	HOMO → LUMO (96%)
	435	0.2304	HOMO-4 → LUMO (95%)
	399	0.2286	HOMO-5 → LUMO (65%)
	344	0.5319	HOMO-4 → LUMO+1 (35%),<br>HOMO → LUMO+3 (33%)
	294	0.2377	HOMO-6 → LUMO+1 (48%)
	279	0.1722	HOMO-5 → LUMO+3 (53%)
OMCD2	597	0.966	HOMO → LUMO (96%)
	518	0.626	HOMO -1 → LUMO (67%)<br>HOMO -4 → LUMO (29%)
	499	0.752	HOMO -4 → LUMO (27%)<br>HOMO -1 → LUMO (28%)
	442	0.782	HOMO → LUMO+1(88%)
	391	0.182	HOMO → LUMO+2(79%)
	345	0.191	HOMO-1 → LUMO+2(36%)<br>HOMO-4 → LUMO+1(35%)
	340	0.407	HOMO-5 → LUMO+1(35%)<br>HOMO-1 → LUMO+2(28%)
	336	0.175	HOMO → LUMO+7(65%)
	323	0.192	HOMO → LUMO+9(43%)<br>HOMO-1 → LUMO+4)
	320	0.207	HOMO → LUMO+9(26%)<br>HOMO-6 → LUMO+1(12%)<br>HOMO-5 → LUMO+1(11%)<br>HOMO-1 → LUMO+3(11%)
	305	0.254	HOMO-7 → LUMO+1(36%)<br>HOMO-6 → LUMO+1(32%)
OMCD3	653	2.155	HOMO → LUMO (96%)
	562	0.541	HOMO → LUMO+1 (93%)
	533	0.142	HOMO-1 → LUMO (88%)
	487	0.672	HOMO-1 → LUMO+1 (78%)
	425	0.921	HOMO → LUMO+2 (59%)<br>HOMO-5 → LUMO+1 (26%)
	422	0.222	HOMO-5 → LUMO+1 (61%)<br>HOMO → LUMO+2 (21%)
	398	0.188	HOMO → LUMO+3 (64%)
	360	0.176	HOMO → LUMO+4 (43%)<br>HOMO → L+5 (25%)
	357	0.190	HOMO-1 → LUMO+3 (78%)

3. Calculated electronic structure and absorption spectra of the adsorbed oligomethine cyanine dyes

NKX-2311

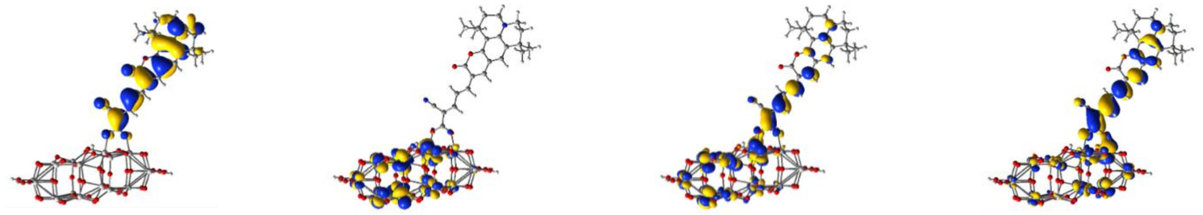

HOMO LUMO LUMO+1 LUMO+2

OMCD1

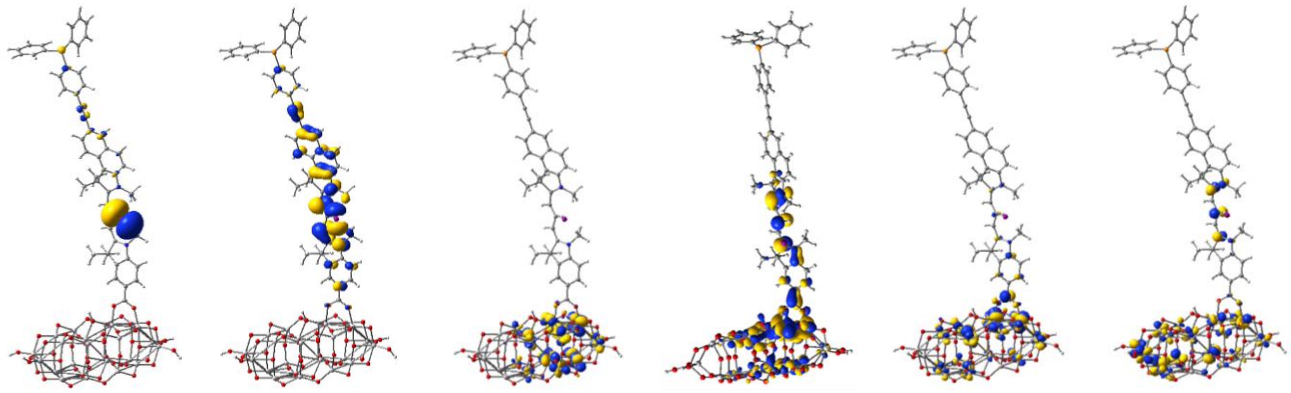

HOMO-3 HOMO LUMO LUMO+1 LUMO+2 LUMO+11

OMCD2

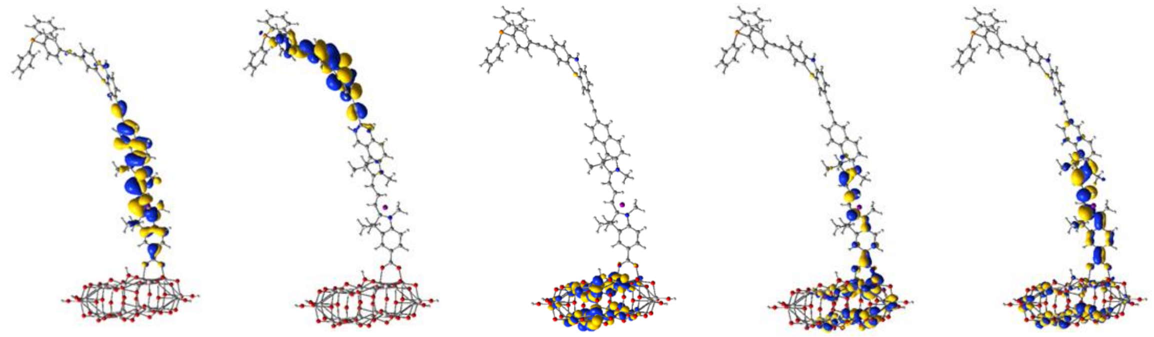

HOMO-1 HOMO LUMO LUMO+4 LUMO+5

OMCD3

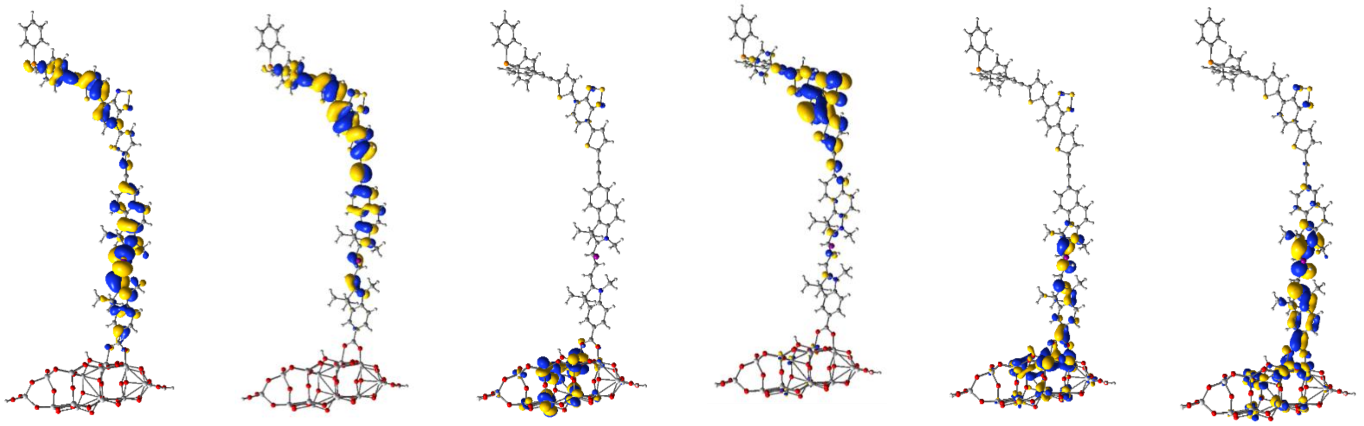

HOMO-1 HOMO LUMO LUMO+1 LUMO+2 LUMO+3

Figure S26. Isodensity surfaces (0.03 e/bohr3) of the key molecular orbitals of NKX-2311, OMCD1, OMCD2, and OMCD3 dyes adsorbed on TiO2 nanocluser, calculated by DFT at the B3LYP/LANL2DZ level in water solvent. Atom colors: T, light grey; C, grey; O, red; N, blue; P, orange; I, purple; and H, small light grey.

Table S3. Contributions of the donor, π-bridge, acceptor groups and the $Ti_{24}O_{50}H_4$ nanocluster to the electron density of the main molecular orbitals of the adsorbed dyes, calculated at the DFT/B3LYP/DZVP level.

Dye	MO	donor unit (%)	π-bridge (%)	acceptor unit (%)	TiO_2 cluster (%)
NKX-2311 /$Ti_{24}O_{50}H_4$	LUMO+4	7	6	4	83
	LUMO+2	16	18	11	55
	LUMO+1	9	10	6	75
	LUMO	0	1	1	98
	HOMO	77	11	12	0
OMCD1 /$Ti_{24}O_{50}H_4$	LUMO+2	0	0	37	63
	LUMO+1	0	0	24	76
	LUMO	0	0	1	99
	HOMO	12	8	80	0
	HOMO-4	50	10	40	0
OMCD2 /$Ti_{24}O_{50}H_4$	LUMO+43	12	27	16	45
	LUMO+5	0	0	48	52
	LUMO+4	0	0	20	80
	LUMO+3	0	0	3	97
	LUMO+1	0	0	2	99
	LUMO	0	0	1	99
	HOMO	7	90	3	0
	HOMO-1	2	9	89	0
OMCD3 /$Ti_{24}O_{50}H_4$	LUMO+3	0	4	50	47
	LUMO+2	0	2	23	75
	LUMO+1	2	87	6	4
	LUMO	0	3	1	95
	HOMO	6	69	24	0
	HOMO-1	8	24	58	0

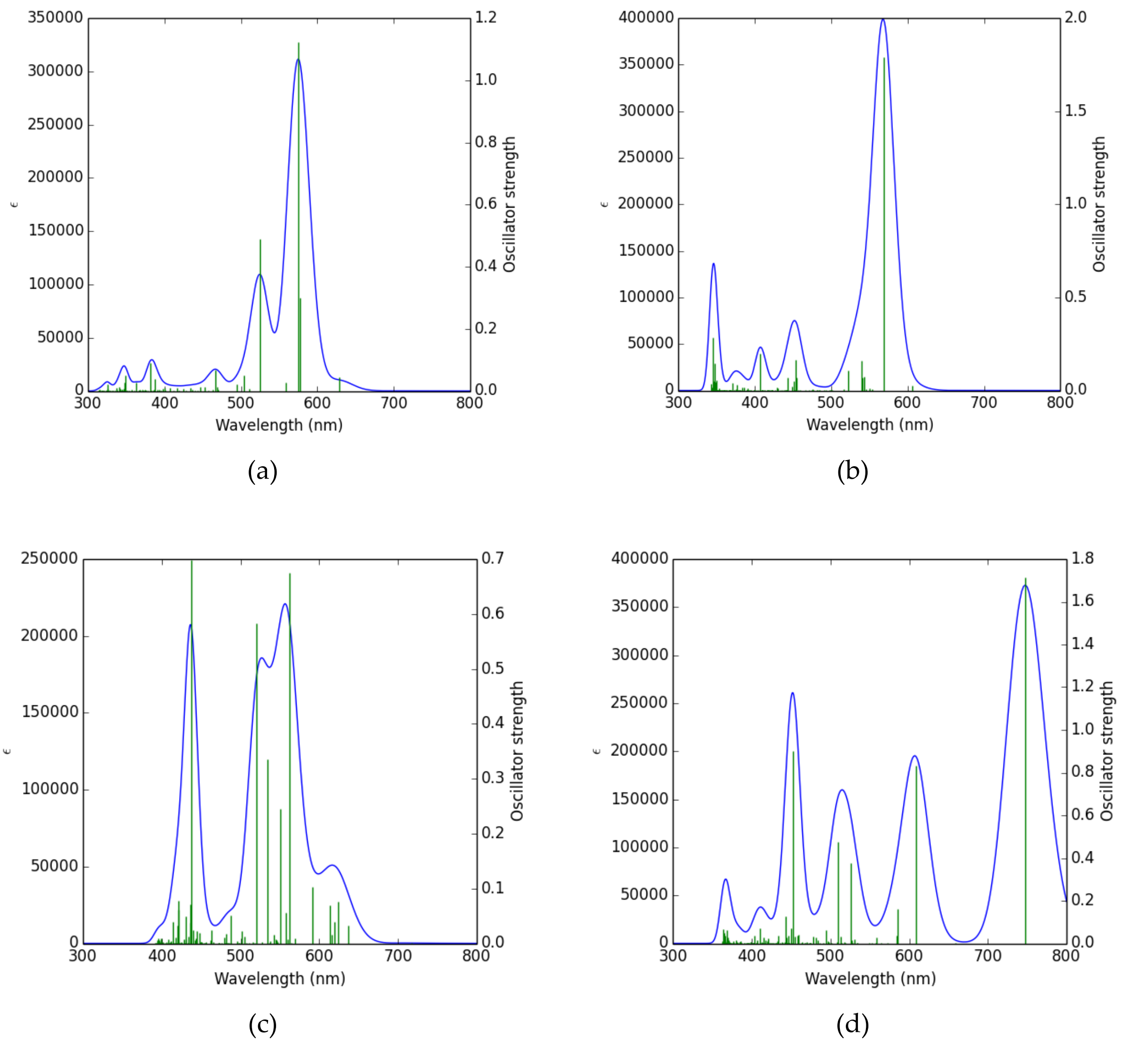

Figure S27. Simulated UV-Vis spectra by DFT calculation for NKX-2311 (**a**), OMCD1 (**b**), OMCD2 (**c**) and OMCD3 (**d**) adsorbed onto $Ti_{24}O_{50}H_4$ nanocluster, in ethanol. The spectral lines were convoluted with Gaussian distributions of 1000 cm-1 linewidth at half maximum.

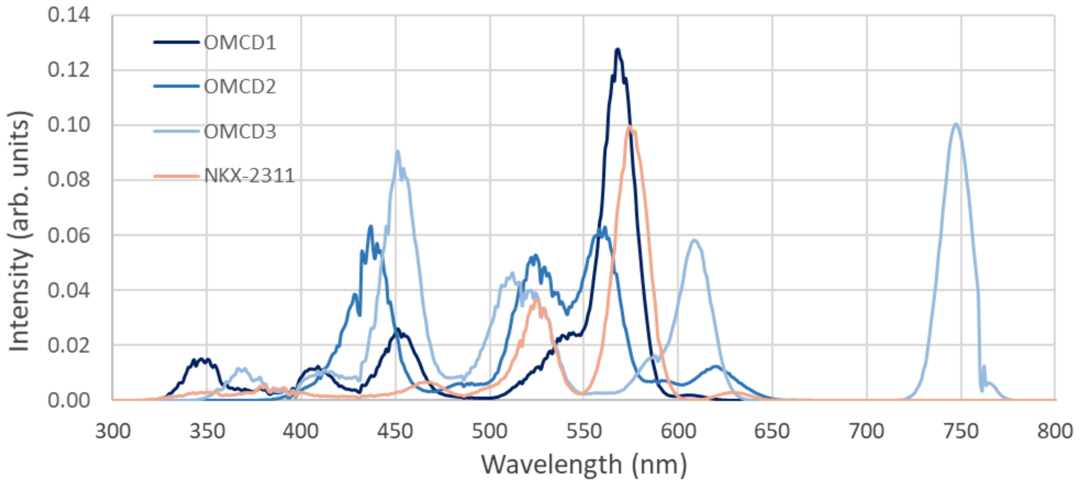

Figure S28. Simulated UV-Vis spectra of the dyes adsorbed onto $Ti_{24}O_{50}H_4$ nanocluster, weighted with the standard solar irradiation.

Table S4 Wavelength, oscillator strength and composition of main electronic transitions of OMCD1, OMCD2 and OMCD 3 adsorbed on $Ti_{24}O_{50}H_4$ cluster

Adsorbed dye	Wavelength (nm)	Oscillator strength	Major contributions
OMCD1 / $Ti_{24}O_{50}H_4$	569	1.790	HOMO → LUMO +1 (55%)
			HOMO → LUMO+2 (28%)
	540	0.160	HOMO -3 → LUMO+1 (17%)
			HOMO -3 → LUMO+2 (16%)
			HOMO -2 → LUMO+1 (12%)
	454	0.147	HOMO-4 → LUMO+1 (41%)
			HOMO-1 → LUMO+6 (31%)
			HOMO-4 → LUMO+2 (12%)
	407	0.196	HOMO-5 → LUMO+1 (33%)
			HOMO-5 → LUMO+2 (26%)
	345	0.283	HOMO-4 → LUMO+25 (25%)
			HOMO-6 → LUMO+3 (18%)
OMCD2 / $Ti_{24}O_{50}H_4$	562	0.675	HOMO-1 →LUMO+1 (37%)
			HOMO-1 → LUMO+4 (17%)
	551	0.246	HOMO-1 → LUMO+1 (52%)
			HOMO-1 →LUMO+4 (16%)
	534	0.335	HOMO-1 → LUMO+4 (30%)
			HOMO-4 →LUMO+5 (26%)
			HOMO-4 → LUMO+4 (20%)
	520	0.583	HOMO-1 → LUMO+5 (63%)
			HOMO-1 → LUMO+4 (14%)
	437	0.670	HOMO → LUMO+43 (37%)
			HOMO → LUMO+44 (17%)
OMCD3 / $Ti_{24}O_{50}H_4$	747	1.716	HOMO → LUMO+1 (93%)
	609	0.833	HOMO → LUMO+2 (54%)
			HOMO → LUMO+3 (32%)
	586	0.163	HOMO-1 → LUMO+1 (59%)
	526	0.378	HOMO-1 → LUMO+2 (46%)
			HOMO -4 → LUMO+1 (31%)
	509	0.477	HOMO-1 → LUMO+3 (43%)
	453	0.903	HOMO → LUMO+29 (36%)
			HOMO → LUMO+30 (17%)
